# Penfluridol targets acid sphingomyelinase to inhibit TNF signaling and is therapeutic against inflammatory autoimmune diseases

**DOI:** 10.1186/s13075-021-02713-6

**Published:** 2022-01-19

**Authors:** Yue-hong Chen, Rong-han Liu, Ya-zhou Cui, Aubryanna Hettinghouse, Wen-yu Fu, Lei Zhang, Chen Zhang, Chuan-ju Liu

**Affiliations:** 1 Department of Orthopaedic Surgery, New York University Grossman School of Medicine, Rm 1608, HJD, 301 East 17th Street, New York, NY 10003 USA; 2 Department of Rheumatology and Immunology, West China Hospital, Sichuan University, Chengdu, 610000 China; 3 Department of Cell Biology, New York University Grossman School of Medicine, New York, NY 10016 USA

**Keywords:** Acid sphingomyelinase, Arthritis, Colitis, Penfluridol, Tumor necrosis factor α, NF-κB

## Abstract

**Background:**

Penfluridol, isolated from an FDA-approved small-molecule drug library as an inhibitor of tumor necrosis factor α (TNFα)-stimulated NF-κB activation, is clinically used to treat chronic schizophrenia and related disorders. This study is aimed to investigate the therapeutic effect of penfluridol on TNFα-stimulated inflammatory autoimmune diseases, particularly inflammatory arthritis.

**Methods:**

Various in vitro studies to confirm the inhibitory effect of penfluridol on TNFα-induced NF-κB activity in bone marrow-derived macrophages or Raw 264.7 macrophage cell line. In vivo studies assessed the therapeutic effects of penfluridol in various disease models, including TNFα transgenic mice, collagen-induced arthritis, DSS-induced colitis, and TNBS-induced colitis. Identification and characterization of the binding of penfluridol to acid sphingomyelinase using bioinformatics and drug affinity responsive target stability assay. Acid sphingomyelinase activity assays to reveal penfluridol-mediated inhibition of acid sphingomyelinase activity. siRNA knockdown experiments to illustrate the dependence of penfluridol’s anti-TNF activity on acid sphingomyelinase.

**Results:**

Penfluridol effectively inhibited TNFα-induced NF-κB activation in vitro and alleviated the severity of arthritis and colitis in vivo. Mechanistic studies revealed that penfluridol bound to acid sphingomyelinase and inhibited its activation. In addition, knockdown of acid sphingomyelinase largely abolished the inhibitory effects of penfluridol on TNFα-induced inflammatory cytokine production. Furthermore, penfluridol suppressed the differentiation of spleen naive CD4+T cells to TH1 and TH17 and inhibited M1 macrophage polarization.

**Conclusion:**

This study provides the rationale for the possible innovative use of penfluridol as a newly identified small-molecule drug for TNFα-driven diseases, such as inflammatory arthritis and colitis.

**Supplementary Information:**

The online version contains supplementary material available at 10.1186/s13075-021-02713-6.

## Introduction

Tumor necrosis factor alpha (TNFα), a pro-inflammatory cytokine released by several cell types such as macrophages and monocytes, plays an important role in normal inflammatory response and immune regulation. However, dysregulation of TNFα-stimulated NF-κB signaling is implicated in wide ranges of diseases, including the autoimmune diseases rheumatoid arthritis (RA) and inflammatory bowel disease (IBD) [1, 2]. Correspondingly, blocking TNFα activity or TNFα-stimulated NF-κB signaling is accepted as an effective treatment strategy [3]. Currently, there are five biologic TNFα inhibitors (TNFi; adalimumab, certolizumab pegol, etanercept, golilmumab, infliximab) which have been approved for clinical use, and the most common application is to treat RA and IBD [4]. Although effective for some patients, TNFi therapy fails to deliver an ACR50 response in up to 50% of RA patients, who need to switch to a second TNFi agent or other biologics [5, 6]. Moreover, adverse effects of TNFi occur in 15% of patients and drug regimens are associated with high cost, approximately $40,000 per year for a patient [6–8].

Development of new drugs that are cheaper and have less adverse effects than TNFi is urgently needed to fill the non-effective treatment gap, though progress is impeded by the extensive cost of bringing a new drug to market, requiring 15 years and $800 million on average [9]. Fortunately, quick screening of FDA-approved on-market small-molecule drug library makes this possible. Through in vitro screening using NF-κB-*bla* THP-1 cell line and in vivo screening using human TNFα transgenic (hTNF-TG):NF-κB-Luc double mutant mice, penfluridol was identified as one of five small-molecule drugs that could inhibit TNFα-stimulated NF-κB activation [10]. Clinically, penfluridol, a first generation diphenylbutylpiperidine antipsychotic with a long half-life of 66 h, is a typical highly potent small-molecule drug used to treat chronic schizophrenia and related disorders [11–14], with treatment efficacy and risk of adverse events similar to chlorpromazine, a benchmark antipsychotic for schizophrenia. Furthermore, penfluridol has a high treatment compliance which benefits from its long half-life [15, 16]. In addition to acting as a typical antipsychotic by targeting D2-like dopamine receptor, penfluridol is also a Ca^2+^-calmodulin inhibitor that inhibits hormonal secretion and decreases growth hormone in a dose-related manner [17]. Here, we report penfluridol is a promising drug for treatment of autoimmune diseases, particularly RA and IBD.

## Materials and methods

### Mice

Both 8-week-old male and female hTNF-TG mice (Taconic Biosciences), male 10–12 week-old DBA/J1 mice (Jackson Laboratory), and male 8-week-old wild-type (WT) C57BL/6 mice (Jackson Laboratory) were used for experiments. Mice, 5 in a cage, were housed in a rodent barrier facility at Skirball Animal Facility of New York University Langone Medical Center on a 12-h light-dark cycle with ad libitum access to food and water. Mice with weight 18–22 g that were in good health condition were enrolled for experiments and no mice were excluded. When starting experiments, animals were randomly assigned to each group on the basis of random number table, six mice were assigned to each group based on empirical choice. All animal experiments were performed in accordance with protocols approved by the Institutional Animal Care and use Committee of New York University School of Medicine.

### Reagents and materials

Dulbecco’s modification of Eagle’s medium (DMEM, 10-017-CM) was purchased from Corning, and fetal bovine serum (FBS, S1620) was bought from Biowest. Human TNFα/TNFSF1A (NFTA0), IL-12 (419-ML-010), TGF-beta (240-B-002) were bought from R&D Systems. Penfluridol (PF, 26864-56-2) and methotrexate (59-05-2) were bought from MedChem Express. Type II chicken collagen (20012), complete Freund’s adjuvant (7001), and incomplete Freund’s adjuvant (7002) were purchased from Chondrex. Dextran sulphate sodium salt (DSS, molecular weight: 36000-50000 Da) was bought from MP Biomedicals. Picrylsulfonic acid solution (Synonym: 2,4,6-Trinitrobenzenesulfonic acid solution, TNBS, 92822), 5-aminosalicylic acid (5-ASA, A-3537), and LPS (L2630) were bought from Sigma-Aldrich. TransAM®NFkB p65 Activation Assay kit (40096) was bought from ACTIVE MOTIF. 3.5-French 38-cm catheter (4193505) was bought from Utah Medical Products, Inc. Plastic feeding tubes, 20 ga × 38 mm (FTP-20-38-50) were bought from INSTECH Laboratories. Antibodies against p-Erk1/2 (4370s), t-Erk1/2 (4695), p-p38 (9211s), t-p38 (9212), p-JNK (9255s), t-JNK (9258), t-p65 (4764s), and GAPDH (2118) were purchased from Cell Signaling Technology. Receptor activator of nuclear factor kappa-B ligand (RANKL, sc-9073) and Lamin B (sc-6216) were bought from Santa Cruz. RNeasy® Mini Kit (74106) and Random Hexamers (79236) were bought from Qiagen. TNFα (PHC3015), mouse IL-1β enzyme-linked immunosorbent assay (ELISA) kit (88-7013), and mouse IL-6 ELISA kit (88706476) were purchased from Invitrogen. VECTASHIELD® Mounting medium with DAPI (H-1200) was bought from Vector Laboratories. SYBR® Green PCR Master Mix (4309155) was bought from Applied Biosystems. ImProm-II^TM^ Reverse Transcriptase (M314C) and Dual-Luciferase reporter assay system (E1910) were purchased from Promega Corporation. Nitrocellulose Membranes (0.45 μm, 162-0115) was bought from Bio-Rad. RIPA Lysis Buffer system (sc-24948A) was bought from ChemCruz. Macrophage Colony-Stimulating Factor (M-CSF, 576406), IFNγ (505702), IL-4 (504102), PE-CD25 (102008), anti-CD3 (100302), anti-CD28 (102102), anti-IL4 (504102), anti-IFNγ (505702), and recombinant mouse IL-6 (575702) were bought from Biolegend. ACK Lysing Buffer (A10492-01) was bought from Gibco. Pronase from *Streptomyces griseus* (PRON-RO) was bought from Roche. Acid Sphingomyelinase Assay Kit (K-3200) was bought from echelon. Antibody against acid SMase/SMPD1 (LS-C334919) was bought from LSBio. Silencer® Select Negative Control siRNA (4390843) was bought from life technologies. TRIzol reagent (15596026), mouse SMPD1 siRNA (151776, AM16708), and NE-PER nuclear and cytoplasmic extraction reagents (78833) were bought from Thermo Fisher Scientific. Fixation/Permeabilization Solution Kit with BD GolgiPlug™ (555028) was bought from BD Biosciences. Mouse Naïve CD4+T cell Isolation Kit (130-104-453) and MACS Separation Columns were bought from Miltenyi Biotec. FITC-CD4 (M1004502) was bought from Sungene. Percp-cy5.5-IFNγ (45-7311-82), PE-IL-17A (12-7177-81), and Alex-Fluo647-FoxP3 (51-5773-80) were bought from eBioscience. PE-IL4 (554389) was bought from BD Pharmingen.

### Bulk RNA-Seq

BMDMs were treated with TNFα (10 ng/mL) in the presence or absence of PF (1μM) for 24 h. Cells were collected to extract total mRNA using RNeasy Plus Mini kit, and mRNA samples were sent to the Genome Technology Center at NYU Langone Medical Center for transcriptome sequencing. RNA-seq transcriptome library was prepared using 1 μg of total RNA. Messenger RNA was isolated based on polyA selection method and then fragmented by fragmentation buffer. Double-stranded cDNA was synthesized and was subjected to end-repair, phosphorylation, and “A” base addition. Libraries were size selected for cDNA target fragments of about 300 bp on 2% Low Range Ultra Agarose followed by PCR amplified for 15 PCR cycles. After quantification by TBS380, paired-end RNA-seq sequencing library was sequenced with the Illumina HiSeq xten sequencer (2 × 150 bp read length).

Those genes downregulated after TNFα stimulation and treatment with penfluridol were used to draw a heatmap and analyze the gene expression differences. We used the online tool TFactS (http://www.tfacts.org/) to identify the transcription factors that regulated those downregulated genes.

### NF-κB luciferase assay

NF-κB luciferase assay was performed according to previously published methods [18]. 293T cells were seeded in 24-well plate and transfected with NF-κB luciferase reporter gene plasmids (1.5 μg) and Renilla plasmids (0.3 μg) by lipofectamine2000 for 8 h. PF was added at concentrations of 0.001, 0.01, 0.1, 1.0, and 2.5μM overnight. The next day, cells were stimulated with TNFα (10 ng/mL) for 4 h. Measurements of luciferase activity were conducted based on the manufacturer’s specifications. Briefly, 100-μL luciferase assay reagents (LARII) were added to wells followed by 20-μL cell lysates and mixture with a pipette tip. Firefly luciferase was measured at a wavelength of 560 nm. After adding 100 μL Stop & Glo® Substrate, a second measurement for Renilla luciferase was performed at 480 nm. The fluorescence results were standardized to Renilla luciferase.

### Western blotting

When inducing BMDMs, freshly isolated cells from the bone marrow were seeded in 6-well or 10-cm plates. BMDMs in 6-well plates were subjected to reduced serum conditions with DMEM supplemented with 2% FBS overnight, followed by adding DMSO or penfluridol (1 μM) for 2 h before stimulating with TNFα (10 ng/mL) for 15, 30, or 60 min. Protein samples were prepared by lysing cells in RIPA buffer containing PMSF, proteinase inhibitor cocktail, and NaVO4. After determining the protein concentrations by bicinchoninic acid (BCA) assay, the proteins were boiled for 5 min in SDS sample buffer. BMDMs in 10-well plates were maintained in DMEM with 2% FBS overnight, followed by adding DMSO or penfluridol (1 μM) for 2 h and then stimulated with TNFα (10 ng/mL) for 15, 30, or 60 min. Cytoplasmic extracts (CE) and nuclear extracts (NE) were prepared using a commercially available kit (NE-PER nuclear and cytoplasmic extraction reagents, 78833, Thermo Fisher Scientific). A total of 20 μg protein samples was separated by gel electrophoresis and transferred to a nitrocellulose membrane using a wet transfer system. The membrane was blocked in 5% (w/v) non-fat milk in TBST for half an hour at room temperature. Incubation with primary antibody was carried out overnight at 4 °C followed by three TBST washes and application of secondary antibody for 1 h at room temperature. The bands on the membrane were developed by enhanced chemiluminescent substrate and visualized by a gel scanner.

### NF-κB DNA-binding activity

BMDMs were treated with PF (1 μM) overnight, then stimulated with TNFα (10 ng/mL) for 4 h. Cells were lysed and NF-κB DNA-binding activity was tested according to the manufacturer’s specifications (TransAM®NFkB p65 Activation Assay kit, 40096, ACTIVE MOTIF). Briefly, NF-κB transcription factor was captured by binding to a consensus sequence 5′-GGGACTTTCC-3′ which was immobilized on a 96-well plate. Nuclear extract (5 μg) was added to each well and incubated with anti-NF-κB p65 antibody, followed by HRP-conjugated secondary antibody incubation. The absorbance was read on a SpectraMax i3x plate reader at a wavelength of 450 nm. Results were expressed as the fold changes of NF-κB DNA-binding activity relative to control cells.

### ELISA

IL-1β and IL-6 levels in cell cultural supernatants or in sera from animal models were measured by mouse ELISA kits based on the manufacturer’s instructions. Optical density was measured by SpectraMax i3x plate reader at a wavelength of 450 nm, and concentrations were calculated according to the standard curve. Supernatants from cells were prepared by addition of TNFα (10 ng/mL) with or without PF (0.1μM or 1 μM) for 24 h.

### Real-time quantitative PCR

Total RNA was extracted from inflamed joints and colons by TRIzol according to the manufacturer’s instructions. To prepare samples, 10-mg tissues were cut into small pieces and put into a grinding tube, then 1 mL TRIzol was added. Grinding of the prepared samples was performed with a tissue homogenizer, a PowerLyzer 24 (Qiagen). Samples were subsequently centrifuged at 10,000*g* for 5 min at room temperature, collecting the supernatant for mRNA extracting.

Total RNA in cells was extracted with RNeasy plus mini kit and cDNA was synthesized using SuperScript® Reverse Transcriptase. SYBR® Green PCR Master Mix was used to perform real-time quantitative PCR (qRT-PCR) on a StepOnePlus^TM^ real-time PCR System (Applied Biosystems). The mRNA expression levels were calculated by ΔΔCT, and fold changes of target mRNA levels were normalized to GAPDH. The following specific sequences as SYBR primers were used for the target gene expansion: mouse IL-1β (5′–3′) F: AAT CTC ACA GCA GCA CAT CA, R: AAG GTG CTC ATG TCC TCA TC; mouse IL-6 (5′–3′) F: TTC CAT CCA GTT GCC TTC TTG, R: AGG TCT GTT GGG AGT GGT ATC; mouse NOS2 (5′–3′) F: TGT TAG AGA CAC TTC TGA GGC TC, R: ACT TTG GAT GGA TTT GAC TTT GAA G; mouse IL-17 (5′–3′) F: GGG AAG TTG GAC CAC CAC AT, R: TTC TCC ACC CGG AAA GTG AA; mouse MCP-1 (5′–3′) F: GTC CCT GTC ATG CTT CTG G, R: GCG TTA ACT GCA TCT GGC T; mouse CXCL10 (5′–3′) F: CCA AGT GCT GCC GTC ATT TTC, R: GGC TCG CAG GGA TGA TTT CAA; mouse GAPDH (5′–3′) F: AGA ACA TCA TCC CTG CAT CC, R: AGT TGC TGT TGA AGT CGC.

### Isolation of bone marrow-derived macrophage

Bone marrow-derived macrophages (BMDMs) were prepared from WT C57BL/6 mice. Following sacrifice, femurs and tibias were collected and cleared of tissue using sterile gauze. Bones were cut to open the medullary cavity and then centrifuged at 13000*g* for 90 s to collect bone marrow cells. Cells were seeded in DMEM containing M-CSF (10 ng/mL), medium was refreshed every third day over a 6-day culture period. Then BMDMs were ready for experiments after stimulation with M-CSF for 6 days.

### hTNFα transgenic (hTNF-TG) mouse model

Eight-week-old hTNF-TG mice, which express a human TNFα gene on a C57BL/6 background and can spontaneously develop arthritis, were used to test the treatment effect of penfluridol on a TNFα-dominant arthritis model [19–21]. Body weight as well as clinical and deformity scores were assessed weekly. Clinical scores were taken as the sums of the scores from digits, paws, wrists, and ankles, and the highest score for each mouse was 24. The detailed scoring systems were as follows: 0 = normal, 0.2 = swollen joint, for 20 digits; 0 = normal, 1 = noticeably swollen, 2 = severely swollen, for 4 paws; 0 = normal, 1 = noticeably swollen, 2 = severely swollen, for 2 wrists; 0 = normal, 2 = noticeably swollen, 4 = severely swollen with stiffness of ankle joint, for 2 ankles. Deformity was ranked on a 0 to 3 scale, corresponding to absence of deformity, mild deformity, moderate deformity, severe deformity, and ankylosis, with the total highest score equaling 12. Prior to administration of drugs, hTNF-TG mice were randomly classified into to three groups (*n* = 6/group): vehicle group, methotrexate group [22] (MTX, 2 mg/kg, a positive treatment control) and penfluridol group (10 mg/kg). MTX and penfluridol were dissolved in DMSO and stored at − 80 °C (for up to 3 months) after aliquoting. For in vivo application, drugs were diluted by a solution of water:ethanol:2% acetic acid at 8:3:1 (v/v), making the final volume of DMSO less than 5%. Drugs were delivered via oral gavage once a day using flexible plastic feeding tubes. For preventative treatment, drugs were given and clinical assessment began on the day when mice were 8 weeks old. For therapeutic treatment, drugs were given and clinical assessment began on the day when the average score was 8. At the end of treatment and observation, mice were sacrificed and sera, knees, and ankles were collected.

### Collagen-induced arthritis model

DBA/1J male mice aged 10–12 weeks were used to establish collagen-induced arthritis (CIA) model [23, 24]. A 100 μL emulsification of type II chicken collagen and complete Freund’s adjuvant was injected intradermally by a 27-G syringe 1.5–2.0 cm away from the tail base. On the 21th day after the first immunization, a 100-μL booster of type II chicken collagen and incomplete Freund’s adjuvant were intradermally given. Clinical scores for erythema and swelling were recorded every other day thereafter until the end of the experiment. The scores were assessed according to the following standards: 0 = normal, 1 = mild swelling involving ankle, wrist, or one digit, 2 = mild swelling involving entire paw or more than two digits, 3 = moderate swelling from the ankle/wrist to entire foot/paw and all digits, 4 = severe swelling or ankylosing deformity of the whole ankle/wrist, foot/paw and digits. The total highest score for a mouse was 16, and a higher score indicated greater severity. Mice were randomly allocated into three groups: vehicle group, MTX (2 mg/kg), and penfluridol (10 mg/kg), with six mice in each group. For prevention groups, daily oral gavage of drugs was initiated on the 18th day after the first immunization while for therapeutic treatment groups, drug delivery began when clinical score had reached an average of 5 in each group. Mice were sacrificed at experimental endpoints, and sera and ankles were collected.

### DSS-induced colitis model

Prior to initiation of dextran sulphate sodium (DSS)-induced colitis, 8-week-old C57BL/6 mice were randomly segregated into vehicle, 5-ASA (50 mg/kg, serving as a positive control), PF 0.4 mg/kg, PF 2 mg/kg, and PF10 mg/kg groups (6 mice in each group). Drugs were delivered via oral gavage once a day until sacrifice starting from 3 days before establishment of the DSS-induced colitis model via addition of 3% DSS drinking water for 5 days and followed by normal drinking water for 3 days [25, 26]. Scores of weight loss, stool consistency, and rectal bleeding were recorded every day beginning on the first day of 3% DSS water supplementation and continuing until sacrifice. Disease activity index was the sum of the scores on weight loss, stool consistency, and rectal bleeding, the higher score correlating to more serious disease activity [27]. The detailed scoring systems were as follows: weight loss (0 = less than 5%, 1 = between 5 and 10%, 2 = between 10 and 15%, 3 = between 15 and 20%, 4 = over 20%), stool consistency (0 = normal, 2 = loose stool, 4 = diarrhea), rectal bleeding (0 = negative, 2 = blood trace, 4 = gross blood). At the end of the experiment, mice were sacrificed, and sera and colons were collected. Colon length was measured by a caliper.

### TNBS-induced colitis model

To establish 2,4,6-trinitrobenzenesulfonic acid (TNBS)-induced colitis model [26], 8-week-old C57BL/6 mice were pre-sensitized by application of 150 μL 1% TNBS on the depilated back between the two forelimbs. Five days later, the mice were randomly assigned to five groups: vehicle, 5-ASA (50 mg/kg), penfluridol 0.4 mg/kg, penfluridol 2 mg/kg, and penfluridol 10 mg/kg, six mice in each group. One week after pre-sensitization, the TNBS-induced colitis model was established by intrarectal injection of 100 μL 3% TNBS. Drugs were supplied by oral gavage one time a day starting from the fifth day after pre-sensitization until the end of experiment. Body weight was recorded every day thereafter. Four days after modeling, mice were sacrificed and colons were collected.

### H&E staining and quantitative analysis

Fresh colons were fixed in 10% formaldehyde for 24 h before processed for paraffin embedding. Sections (5μm) were cut from tissue blocks and went through deparaffinized, rehydrated, routine H&E staining, dehydrated, and cleared for mounting.

H&E-stained sections of ankle or knee joints were used to assess the severity of arthritis via scoring on the basis of inflammation, pannus formation, and cartilage damage [28]. The inflammation scores were 0, 1, and 2 corresponding to normal, local inflammatory cell infiltration, obvious inflammatory cell infiltration that formed lymphoid aggregates, and edema, respectively. Scores for pannus formation and cartilage damage were as follows: 0 = normal, 1 = pannus formation without articular cartilage infiltration, 2 = pannus formation with articular cartilage infiltration.

H&E staining score of colons [29]: the scores for colitis were the sum scores of inflammatory cell infiltration and intestinal wall structure integrity, the higher scores indicated more serious inflammation. For scores of inflammatory cell infiltration: 0 = normal, 1 = inflammatory cell only infiltrated to mucosa, 2 = inflammatory cell infiltrated to mucosa and sub-mucosa, 3 = inflammatory cell was found in the whole intestinal wall. Scores for intestinal wall structure integrity were assessed by the change of epithelial cell: 0 = normal, 1 = inflammatory cell was locally infiltrated, 2 = focally formed ulceration, 3 = extensively formed ulceration with or without granulation tissue or pseudo-polyps.

### Prediction of penfluridol binding target

Two steps were needed to predict the binding target of penfluridol. Firstly, finding out the canonical SMILES of penfluridol by visiting the online website, https://www. ncbi.nlm.nih.gov/pccompound. Secondly, visiting the prediction website (http://www.swisstargetprediction.ch/) [30], an open website for drug target prediction, to find the possible binding target of penfluridol. The prediction result showed that the acid sphingomyelinase (ASM) was the target of penfluridol.

### DARTs

Drug affinity responsive target stability (DARTs) experiments were performed to determine the binding between penfluridol and ASM based on previous reports [31, 32]. Cells were lysed by lysis buffer (1× protease inhibitor cocktail, 50 mM sodium fluoride, 10 mM β-glycerophosphate, 5 mM sodium pyrophosphate, 2 mM sodium orthovanadate, 69% M-PER, V: V) on ice for 10 min. Lysates were collected into a sterile pre-chilled 1.5-mL tube and centrifuged at 18,000*g* for 10 min at 4 °C, and the supernatant was transferred to a new 1.5-mL tube. After measuring the protein concentration by BCA assay, 99-μL cell lysates were mixed with 1 μL DMSO or 1 μL penfluridol (1 mM) and incubated with shaking for 30 min at room temperature. Cell lysate mixtures were then divided into 20-μL aliquots for addition of pronase at a concentration of 1:800 or 1:1600 (pronase: total protein). Digestion was carried out for 5 min at room temperature and stopped by addition of 20× protease inhibitor cocktail and incubation on ice for 10 min. Following digestion, samples were mixed with SDS-PAGE loading buffer and boiled for 5 min at 100 °C. The samples were used to perform western blotting and ASM target bands were visualized by enhanced chemiluminescent substrate using a gel scanner.

### Acid sphingomyelinase activity

Assays for ASM activity were performed according to the manufacturer’s specifications. Briefly, Raw 264.7 cells at 70–80% confluence were treated with or without TNFα (10 ng/mL) overnight. After washing twice with phosphate-buffered saline, cells were collected into a 1.5-mL tube in cell collection buffer by cell scraper. Then cells were lysed by freeze-thaw in liquid nitrogen for three cycles, and cell lysates were centrifuged at 14,000 rpm at 4° for 10 min. Supernatants were collected, and protein concentration was detected by BCA assay and was adjusted to 1.5 mg/mL. In total, 20 μL of cell lysates was added to a 96-well plate in the presence of varied concentrations (0.1, 1, 10, 100 μM) of penfluridol and incubated on a shaker for 1 h at room temperature. The following steps were on the basis of the manufacturer’s specifications. Briefly, after incubating cell lysates with ASM substrate for 3 h at 37 °C and adding stop buffer, the plate was read at 360 ex/460 em. The activity of ASM in each group was calculated on the basis of standards.

### Spleen naïve CD4+T cell differentiation

Induction of spleen naïve CD4+T cell differentiation into T cell subsets was performed according to previously reported methods [33, 34]. One day before cell differentiation induction, 1 mL PBS containing anti-CD3 (1 μg/mL) and anti-CD28 (1 μg/mL) was coated for TH1, TH2, and Treg and 1 mL PBS containing anti-CD3 (2 μg/mL) and anti-CD28 (1 μg/mL) was coated for TH17 in a 24-well plate and the plate was incubated at 4 °C overnight. The next day, spleens were isolated from WT C57BL/6 mice and spleen cells were isolated by grinding spleen on a 70-μm mesh and collected in PBS containing 1% FBS, then centrifuged at 1000 rmp for 5 min twice, followed by lysing red blood cells with ACK lysing buffer for 5 min on ice. After centrifugation, naïve CD4+T cells were isolated by CD4+T cell isolation kit in MACS separation columns according to the manufacturer’s specifications. Coating buffer was aspirated, and the plate was washed once with PBS prior to seeding CD4+T cells (1 × 10^6^ per well) in 1.0 mL DMEM. The following cytokines were added to induce T cell differentiation: IL-2 (20 ng/mL), IL-12 (15 ng/mL), and anti-IL4 (5 μg/mL) for TH1; IL-2 (20 ng/mL), IL-4 (10 ng/mL), and anti-IFN γ (5 μg/mL) for TH2; IL-6 (20 ng/mL), TGFβ (3 ng/mL), anti-IFN γ (5 μg/mL), and anti-IL4 (5 μg/mL) for TH17; IL-2 (20 ng/mL), TGFβ (15 ng/mL), anti-IFN γ (5 μg/mL), and anti-IL4 (5 μg/mL) for Treg. At the same time, penfluridol (0.1μM) or penfluridol (1μM) were added. Four days later, cells were collected for flow cytometry analysis.

To test the differentiation, 1 μL Golgi stop buffer was added to each well for 4 h. T cell subset stain was based on the specfications of Fixation/Permeabilization Solution Kit. TH1 were stained with FITC-CD4 and Percp-cy5.5-IFNγ, TH2 were stained with FITC-CD4, PE-IL4, TH17 were stained with FITC-CD4 and PE-IL-17A, and Treg were stained with FITC-CD4, PE-CD25, and Alex-Fluo647-FoxP3. For intracellular staining, antibodies were diluted at 1:100 while, for cell surface staining, antibodies were diluted at 1:400. All samples were sent to NYU Langone Medical Center for detecting.

### Statistical analysis

Assessment of clinical signs, colon length measurement, and histology scoring were performed by a pair of two investigators. Blinding was applied to one of the two investigators for the whole data collection process but not for data analysis. All data were organized and analyzed in GraphPad 8.0 and SPSS 22.0 statistics software. Data were expressed as mean with standard error of the mean. Statistical significance of the differences among groups was determined by one-way analysis of variance and Bonferroni post hoc test. The statistical differences of clinical scores among different groups were analyzed by repeated measures analysis of variance. *p* < 0.05 was considered as statistically significant.

## Results

### Penfluridol is isolated as an anti-NF-κB activation drug

**Fig. 1:**
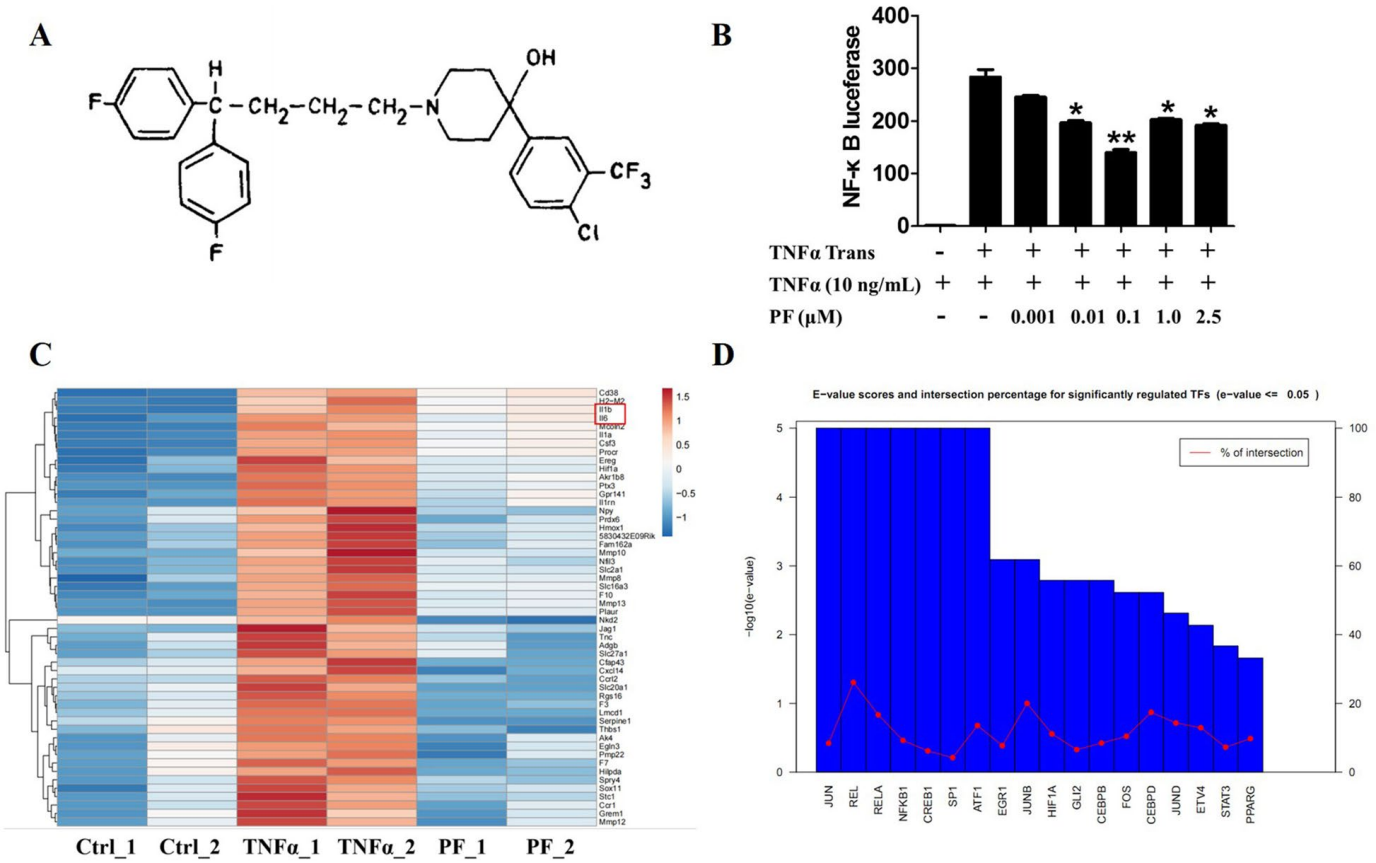
Penfluridol is isolated as an anti-NF-κB activation drug. Penfluridol was identified as an anti-NF-κB activation drug stimulated by TNFα from a FDA-approved small-molecule drug library. NF-κB luciferase assay and bulk RNA-seq were used to confirm the inhibitory effect of penfluridol on TNFα-induced NF-κB activity. **A** The molecular structure of penfluridol (PF). **B** NF-κB luciferase assay of penfluridol at different doses in 293T. **C** Heat map analysis of down expression genes regulated by penfluridol after TNFα stimulation (*n* = 2). Penfluridol decreases a wide range of TNFα-induced gene expressions, including IL-1β and IL-6 indicated by a red rectangle. **D** Transcription factor enrichment analysis of RNA-Seq results. Penfluridol regulates a wide range of transcription factors, including RelA P65 and NF-κB1 P105. Experiments were performed for 3 biological replications, and one-way analysis of variance and Bonferroni post hoc test were used to test statistical significance of the differences among groups (*p < 0.05, **p < 0.01). PF: penfluridol

Through first screening a FDA-approved small-molecule drug library containing 1046 drugs using NF-κB-*bla* THP1, secondary confirmation by NF-κB luciferase reporter assay, and third in vivo verification in hTNF-TG/NF-κB luc mutant mice, we identified penfluridol as one of five drugs that could inhibit TNFα-induced NF-κB activity [10]. Penfluridol is clinically used as an antipsychotic drug to treat various types of schizophrenia and its molecular structure is presented (Fig. 1A). To further confirm the inhibitory effect of penfluridol on TNFα-induced NF-κB activity, NF-κB luciferase assay was performed in the presence of varied concentrations of penfluridol and results illustrated that penfluridol dose-dependently reduced TNFα-stimulated NF-κB activity (Fig. 1B). We also used bulk RNA-Seq to verify the inhibitory effect of penfluridol on TNF signaling downstream gene expressions after the activation of NF-κB by TNFα. RNA-Seq results showed penfluridol decreased expressions of about 50 genes induced by TNFα, including chemokine family members (such as CCR1, CCR12, CXCL14), interleukins (such as IL-1α, IL-1β, IL-6), and matrix metalloproteinases (such as MMP8, MMP10, MMP12, MMP13), which are organized in the heatmap (Fig. 1C) and listed in the supplemental Table 1. We used the online tool TFactS (http://www.tfacts.org/) to identify the transcription factors that regulated these downregulated genes and found transcription factors RelA P65 and NF-κB1 P105 were involved in the regulation of these genes which could be obviously regulated by penfluridol (Fig. 1D).

### Penfluridol inhibits TNFα-induced NF-κB activity in vitro

**Fig. 2:**
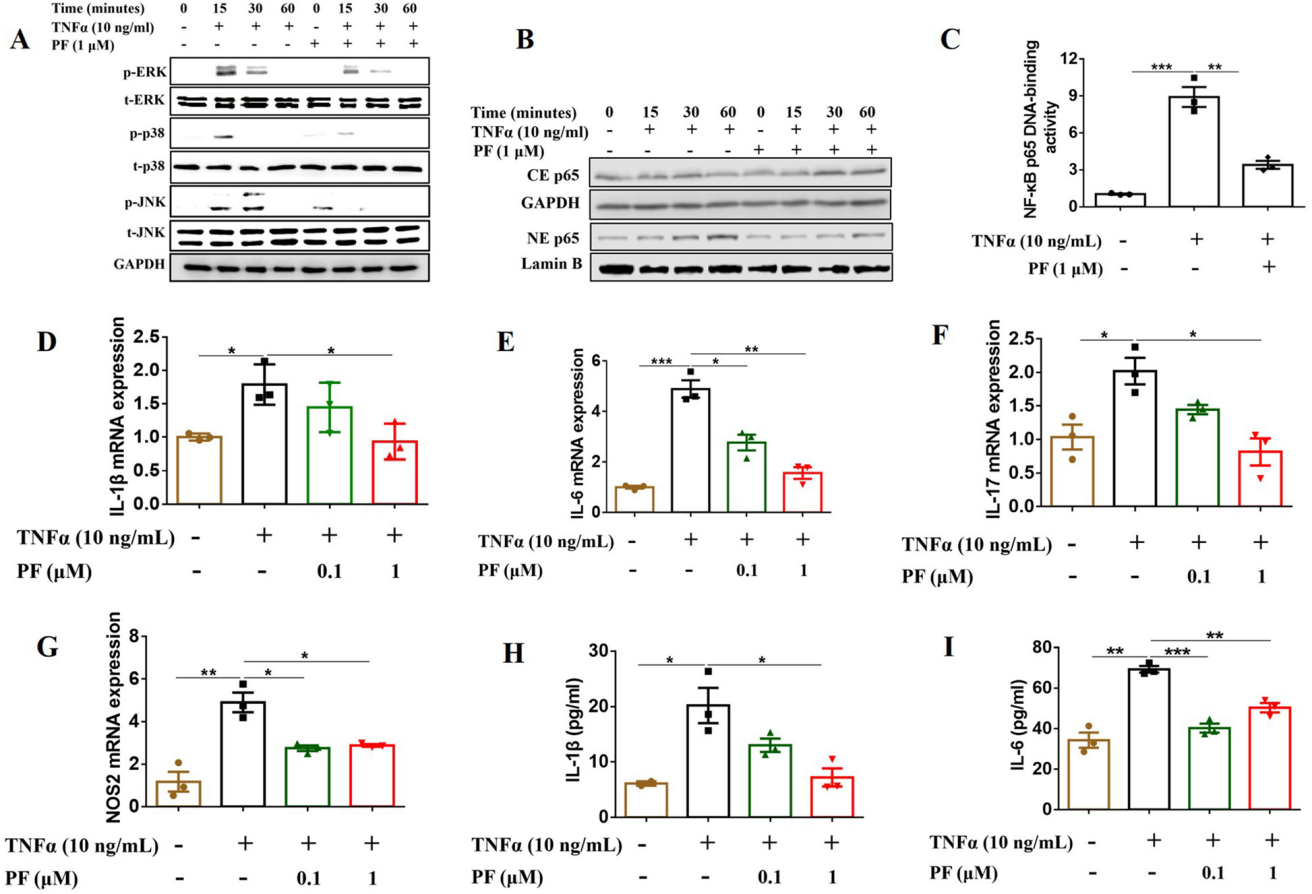
Penfluridol inhibits TNFα-induced NF-κB activation in vitro. BMDMs were starved with 2% FBS overnight, followed by adding DMSO or penfluridol (1 μM) for 2 h and then stimulated with TNFα (10 ng/mL) for 0, 15, 30, or 60 min. **A** Western blotting to test the effect of penfluridol on the phosphorylation of MAPKs induced by TNFα in BMDM isolated from WT C57BL/6 mice. **B** Western blot to determine the p65 translocation from cytoplasm to nucleus in BMDM. **C** After starving BMDMs with 2% FBS overnight, the cells were treated with DMSO or penfluridol (1 μM) for 2 h, followed by stimulation with TNFα (10 ng/mL) for 4 h, then nuclear extracts were used to test the binding activity of p65 to DNA in BMDM, detected by ELISA. **D–I** After adding DMSO or penfluridol (1 μM) for 2 h, BMDMs were stimulated with TNFα (10 ng/mL) for 24 h, then supernatants were collected to detect cytokine secretion levels by ELISA and cells were collected to test mRNA expression levels of cytokines by real-time quantitative PCR (qRT-PCR). **D** mRNA expression level of IL-1β. **E** mRNA expression level of IL-6. **F** mRNA expression level of IL-17. **G** mRNA expression level of NOS2. **H** Secretion level of IL-1β. **I** Secretion level of IL-6. Experiments were performed for 3 biological replications, and one-way analysis of variance and Bonferroni post hoc test were used to test statistical significance of the differences among groups (*p < 0.05, **p < 0.01, ***p < 0.001). PF: penfluridol

To further verify the inhibitory effect of penfluridol on TNFα-stimulated NF-κB activation in vitro, we assessed the phosphorylation of several molecules in the NF-κB signaling pathway, translocation of p65, binding activity of NF-κB p65 to DNA, and mRNA expression and release levels of several inflammatory cytokines in BMDMs or Raw 264.7 cells. When stimulating BMDMs with TNFα (10 ng/mL), the phosphorylation levels of ERK, JNK, and p38 were significantly increased which were obviously inhibited by penfluridol (1 μM) (Fig. 2A, Sfig. 1A-C). When stimulating BMDMs with TNFα (10 ng/mL), p65 translocated from cytoplasm to nuclei, which was inhibited by penfluridol (1 μM) (Fig. 2B, Sfig. 1D-E). In addition, penfluridol reduced TNFα-induced binding activity of p65 to DNA (Fig. 2C). qRT-PCR and ELISA assays further showed that penfluridol inhibited TNFα-induced mRNA expressions of IL-1β, IL-6, IL-17, and NOS2 (Fig. 2D–G) and suppressed the cytokine secretion levels of IL-1β and IL-6 (Fig. 2H,I) in BMDMs isolated from WT C57BL/6 mice. The inhibitory effects of penfluridol on TNFα-induced mRNA expression and secretion of inflammatory cytokines were also observed in macrophage cell line Raw 264.7 cells (Sfig. 2 A-F) and BMDMs isolated from hTNF-TG mice (Sfig. 3 A-E).

As NF-κB activity stimulated by TNFα also plays a pivotal role in osteoclastogenesis, the effect of penfluridol on osteoclastogenesis was examined and results revealed that penfluridol could markedly inhibit TNFα-enhanced osteoclastogenesis (Sfig. 4 A-B). Penfluridol inhibited the production of inflammatory cytokines, such as TNFα, IL-1β, and IL-6, which are involved in regulating macrophage polarization [35]; accordingly, we also investigated whether penfluridol affected macrophage polarization. M1 macrophages were pro-inflammatory macrophages and secreted a wide range of inflammatory cytokines such as TNFα, IL-1β, and IL-6 while M2 macrophages are anti-inflammatory macrophages, releasing anti-inflammatory cytokines like IL-10. Therefore, IL-6 and NOS2 were chosen as the markers for M1 macrophages while Arg1 and Mgl1 as the markers for M2 macrophages. The results suggested that penfluridol inhibited M1 macrophage polarization evidenced by decreased mRNA expression levels of IL-6 and NOS2, but had no effect on M2 macrophage polarization evidenced by no alteration in M2 markers, Arg1 and Mg11 (Sfig. 4 C-F).

### Penfluridol prevents the onset and severity of arthritis in hTNF-TG mice

**Fig. 3:**
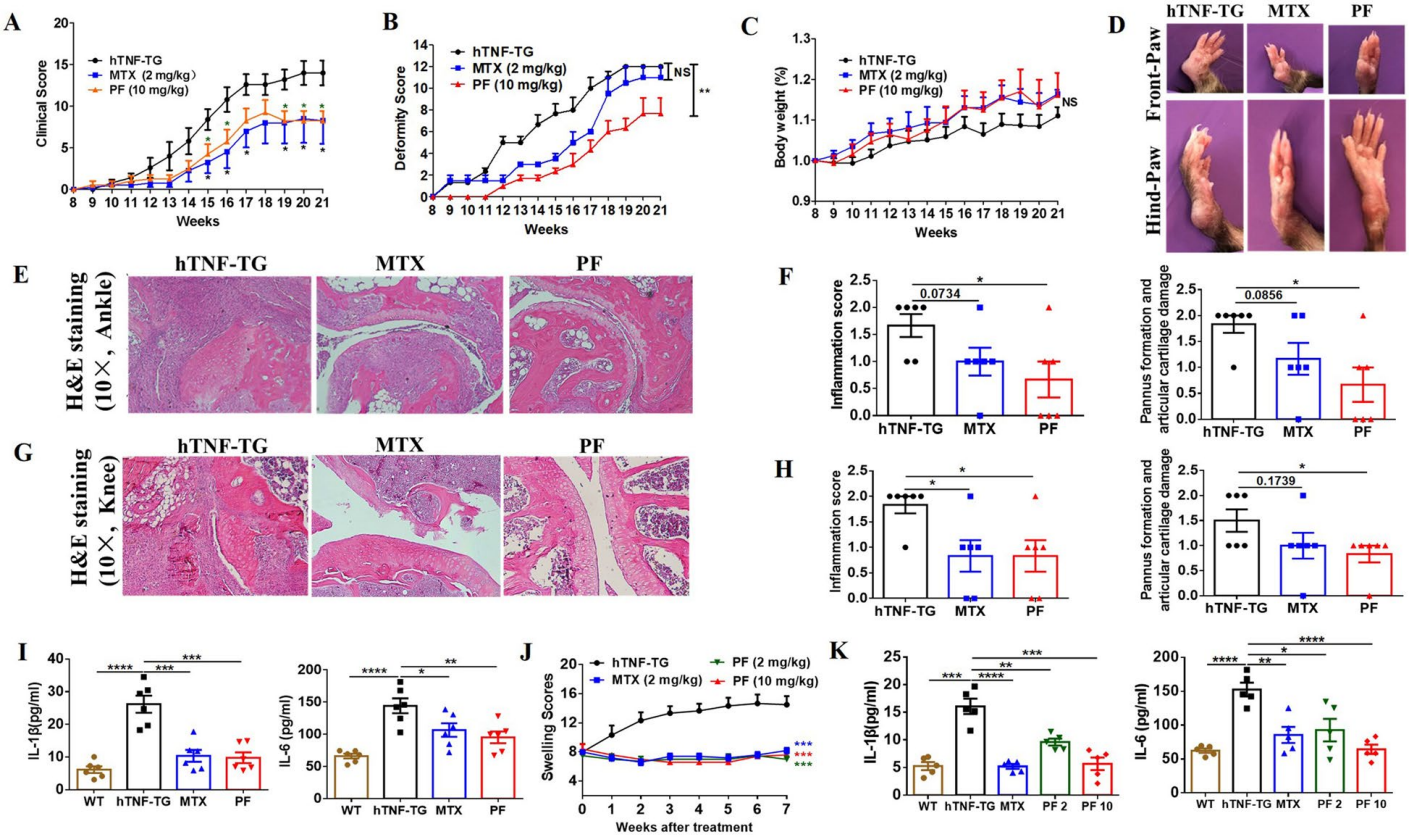
Penfluridol prevents the onset and severity of arthritis in hTNF-TG mice. TNFα overexpression mouse model (hTNF-TG) which can spontaneously develop arthritis were used to observe the inhibitory effect of penfluridol on TNFα-induced NF-κB activation in vivo. Drugs were administrated by oral gavage once a day. Prevention treatment is penfluridol was given to mice at the eighth week of age when arthritis symptom began its onset; treatment of penfluridol was administrated when average clinical arthritis scores reached 8 points in each group. **A** Clinical score of prevention treatment. **B** Deformity score of prevention treatment. **C** Body weight of prevention treatment. **D** Representative pictures of both front and hind paws in each prevention treatment groups. **E** H&E staining of paraffin-embedded ankle slides in prevention treatment groups. **F** Quantitative analysis of inflammation score and pannus formation and cartilage damage score of **E**. **G** H&E staining of paraffin-embedded knee sections in preventive treatment groups. **H** Quantitative analysis of inflammation score and pannus formation and cartilage damage score of **G**. **I** Serum IL-1β and IL-6 levels in preventive treatment groups, detected by ELISA. **J** Clinical scores in treatment groups. **K** Serum levels IL-1β and IL-6 in treatment groups, detected by ELISA. Six mice in each group. One-way analysis of variance and Bonferroni post hoc test were used to test statistical significance of the differences among groups, and repeated measures analysis of variance was used to test statistical differences of clinical scores among different groups (*p < 0.05, **p < 0.01, ***p < 0.001, ****p < 0.0001). PF: penfluridol

The inhibitory effect on TNFα-induced NF-κB activation was confirmed in vitro, which prompted us to investigate the treatment effect of penfluridol in vivo in TNFα-dominant disease. hTNF-TG mice overexpressing human TNFα and spontaneously developing arthritis were used to test the treatment effect of penfluridol. Arthritis onset began approximately at 8 weeks of age with severity increasing longitudinally. Preventative treatment with penfluridol began at the 8 week of age to prevent progression of arthritis and weekly assessment of clinical signs coincided with commencement treatment. MTX served as the positive control as it is a small-molecule drug that is the first line treatment for RA in clinic. Results showed that penfluridol could delay arthritis onset time and alleviate arthritis severity, with similar efficacy as the positive control drug MTX (Fig. 3A). As for the joint deformity, the treatment effect of penfluridol was superior to MTX, as demonstrated by the fact penfluridol could delay the deformity onset time and alleviate severity of joint deformity (Fig. 3B, D). Moreover, penfluridol did not affect body weight (Fig. 3C). H&E staining of ankle and knee sections suggested penfluridol reduced inflammatory cell infiltration, inhibited pannus formation, and protected cartilage from damage (Fig. 3E–H). To determine whether the reduced inflammatory cell infiltration and alleviated severity of joint inflammation were attributable to the inhibitory effect of penfluridol on TNFα-stimulated NF-κB activation, we tested downstream cytokines of NF-κB by qRT-PCR and ELISA. mRNA expression levels of chemokines CXCL10 and MCP-1 (Sfig. 5 A-B) in the inflamed joints and the production and release levels of cytokines IL-1β and IL-6 in the sera were significantly reduced after treatment of penflurdol (Fig. 3I). In sum, penfluridol had a prophylactic effect in the TNFα-dominant arthritis.

Further, we tested whether penfluridol had treatment effect to prevent disease progression in the TNFα-dominant arthritis. Penfluridol was administered in the hTNF-TG mice when average clinical arthritis scores reached 8 points in each group. Results showed penfluridol could prevent disease progression (Fig. 3J) and reduce the production of inflammatory cytokines L-1β and IL-6 in a dose-dependent manner (Fig. 3K). Collectively, penfluridol was effective in treatment of TNFα-dominant arthritis.

### Penfluridol alleviates the severity of collagen-induced arthritis

**Fig. 4:**
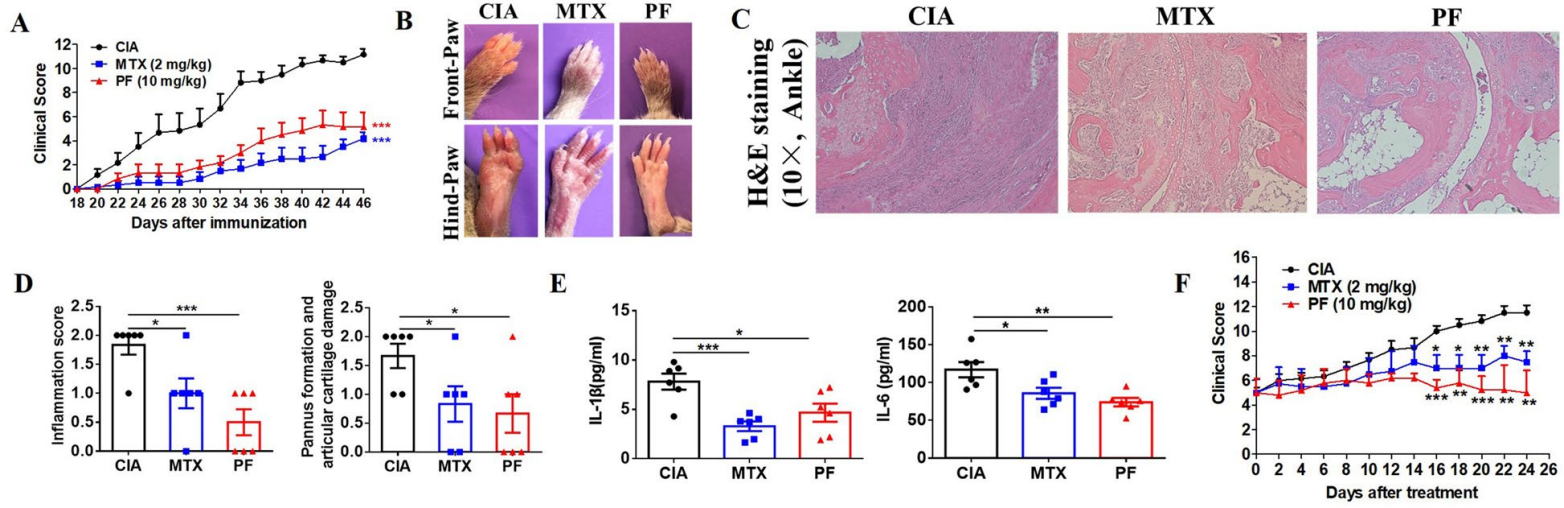
Penfluridol alleviates severity of collagen-induced arthritis. Type II chicken collagen-induced arthritis (CIA) model were adopted to assess the prevention and treatment effect of penfluridol in NF-κB-activated arthritis and drugs were given one time a day by oral gavage. Preventive treatment was administrating penfluridol starting from the 18th day after the first immunization while therapeutic treatment was administrating penfluridol when clinical score reached an average of 5 in each group. **A** Clinical score in preventive treatment. **B** Representative pictures of both front and hind paws in preventive treatment groups. **C** H&E staining of paraffin-embedded ankle slides in preventive treatment groups. **D** Quantitative analysis of inflammation score and pannus formation and cartilage damage score of **C**. **E** Serum levels of IL-1β and IL-6 levels in preventive treatment groups, detected by ELISA. **F** Clinical scores in treatment groups. Six mice in each group. One-way analysis of variance and Bonferroni post hoc test were used to test statistical significance of the differences among groups (*p < 0.05, **p < 0.01, ***p < 0.001). PF: penfluridol

The effective preventive and therapeutic effects of penfluridol in TNFα-dominant mouse model prompted us to test whether penfluridol had the same therapeutic effect in the diseases in which pathogenesis was dominant by TNFα and TNFα-stimulated NF-κB activation, such as RA and IBD [2]. CIA mouse model, the most commonly used mouse model to imitate the pathogenesis of RA, was used to test the treatment effect of penfluridol in NF-κB-activated arthritis. In our preventative treatment scheme, penfluridol was administrated starting from the 18th day after the first immunization before the disease onset. In a separate therapeutic treatment scheme, penfluridol was administrated when clinical score had reached an average of 5 in each group. Results of preventative treatment demonstrated penfluridol was associated with significantly reduced severity of arthritis, which was comparable to the firstline treatment MTX (Fig. 4A,B). The H&E staining of ankles suggested that penfluridol inhibited inflammatory cell infiltration, suppressed pannus formation, and protected articular cartilage from damage (Fig. 4C,D). To further characterize the inhibitory effect of penfluridol on NF-κB activation, downstream cytokines of NF-κB were detected by qRT-PCR and ELISA. Results showed mRNA expressions of CXCL10 and MCP-1 were obviously decreased in inflamed joints (Sfig. 5 C-D) and production levels of inflammatory cytokines IL-1β and IL-6 were statistically reduced in sera after penfluridol treatment (Fig. 4E). Results of therapeutic effects suggested penfluridol was associated with significantly reduced clinical scores, which was comparable to MTX treatment (Fig. 4F). In short, penfluridol was effective to prevent and treat CIA.

### Penfluridol is therapeutic against IBD mouse models

**Fig. 5:**
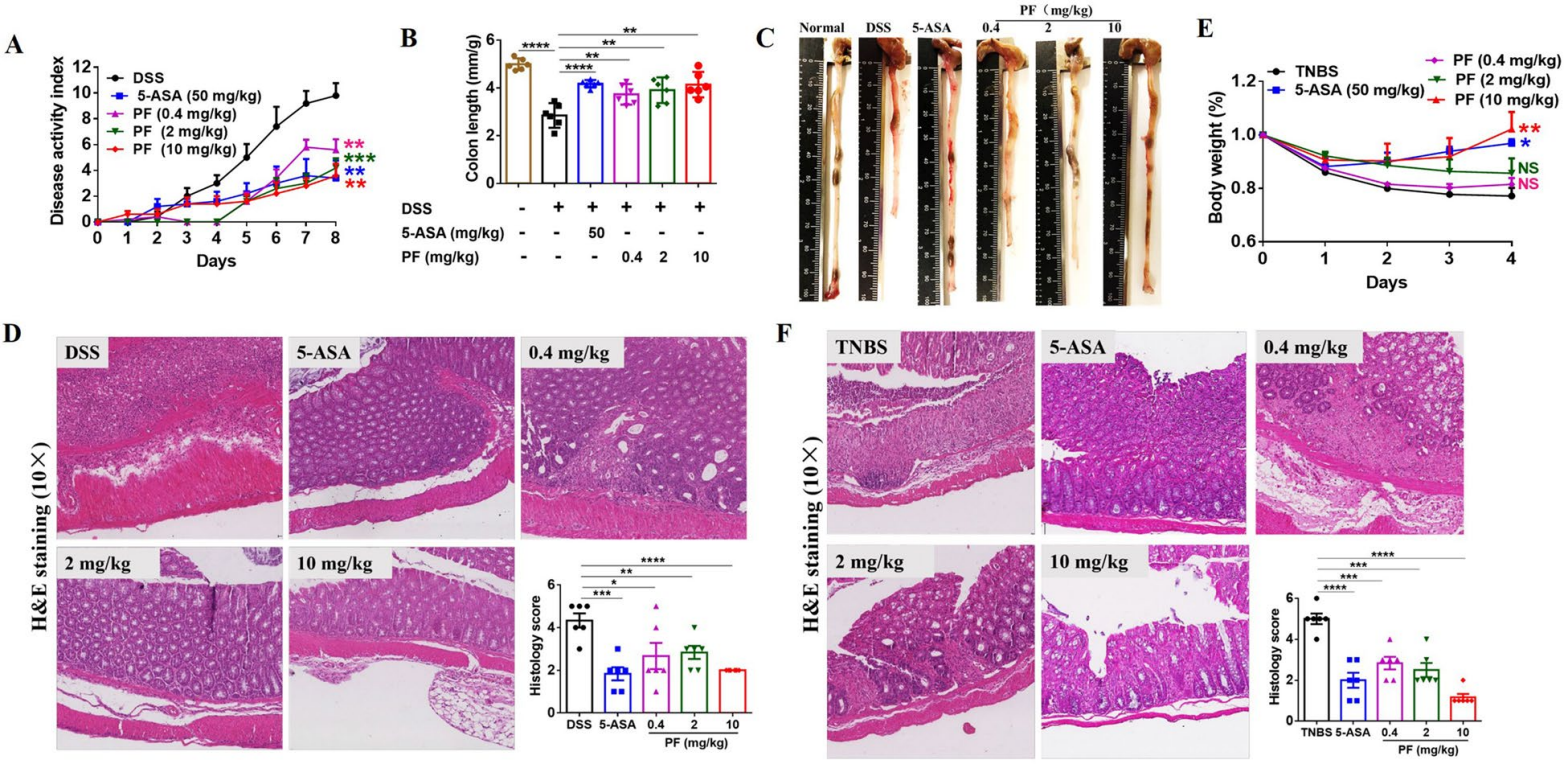
Penfluridol is therapeutic against IBD mouse models. DSS-induced colitis and TNBS-induced colitis model were established to assess the treatment effect of penfluridol in NF-κB-activated colitis in which drugs were given once a day by oral gavage. **A** Disease activity index of DSS-induced colitis model. **B** Colon length of DSS-induced colitis model. **C** Representative colon length in each group of DSS-induced colitis model. **D** H&E staining of colon tissues and quantitative analysis of H&E staining of DSS-induced colitis model. **E** Body weight of TNBS-induced colitis model. **F** H&E staining of colon tissues and quantitative analysis of H&E staining of TNBS-induced colitis model. Six mice in each group. One-way analysis of variance and Bonferroni post hoc test were used to test statistical significance of the differences among groups, and repeated measures analysis of variance was used to test statistical differences of clinical scores among different groups (NS = not significance, *p < 0.05, **p < 0.01, ***p < 0.001, ****p < 0.0001). PF: penfluridol

As penfluridol could inhibit TNFα-induced NF-κB activity and was effective to alleviate the severity of TNFα and TNFα-stimulated NF-κB activation-driven arthritis, we assessed the treatment efficacy of penfluridol in another TNFα-dominant autoimmune disease, IBD. The DSS-induced colitis mouse model is a model which mimics ulcerative colitis (UC). Penfluridol treatment was initiated 3 days ahead of model establishment and 5-ASA was chosen as the positive control considering it is a small-molecule drug and is used as a firstline treatment. Penfluridol, at three doses, statistically reduced disease activity index to a degree comparable with the positive control drug 5-ASA (Fig. 5A) and was found to be effective in preventing shortening of colon length, the feature of UC (Fig. 5B,C). H&E staining of the colons showed penfluridol was as effective as positive control drug 5-ASA to inhibit inflammatory cell infiltration and protect the integrity of the intestinal wall (Fig. 5D). The downstream molecules of NF-κB activity were detected by qRT-PCR and results showed mRNA expression levels of CXCL10 and MCP-1 were decreased in penfluridol-treated colon tissues (Sfig. 5 E-F).

We also tested the treatment effect of penfluridol in the TNBS-induced colitis mouse model, which resembles human Crohn’s disease (CD). Penfluridol was administrated 5 days later after pre-sensitized of 1% TNBS. High-dose (10 mg/kg) penfluridol prevented body weight loss and was as effective as positive control drug 5-ASA while lower doses of penfluridol did not appreciably prevent body weight loss (Fig. 5E). H&E staining of colons revealed that each tested dose of penfluridol was effective to inhibit inflammatory cell infiltration and protect the integrity of intestinal wall (Fig. 5F). The downstream molecules of NF-κB activity were detected by qRT-PCR, and results reported mRNA expression levels of CXCL10 and MCP-1 were obviously decreased in penfluridol-treated colon tissues (Sfig. 5 G-H). Collectively, penfluridol was therapeutic against IBD including UC and CD.

### Acid sphigomyelinase is a target of penfluridol

**Fig. 6:**
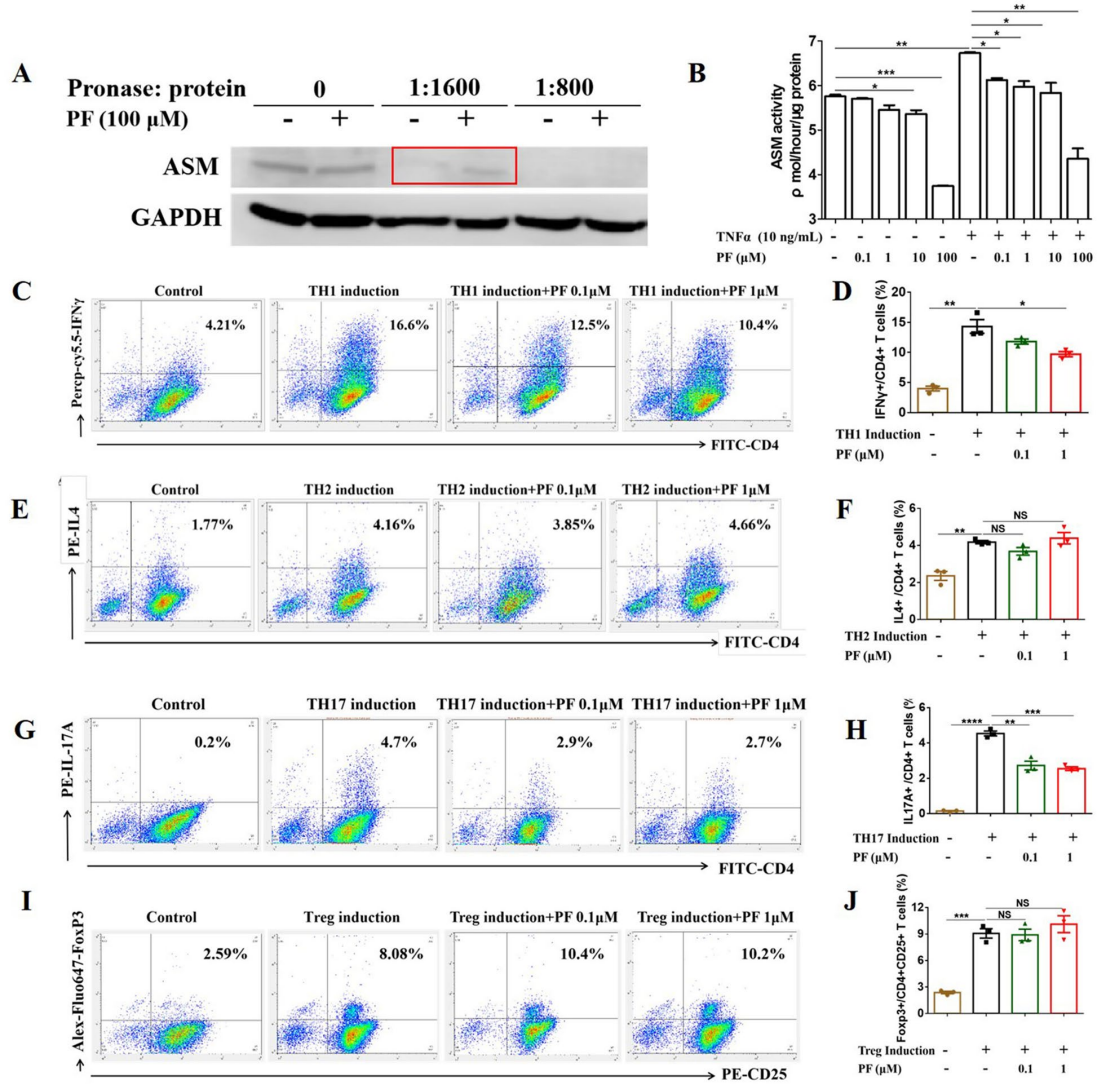
Acid sphigomyelinase is a target of penfluridol. **A** Raw 264.7 cells were collected to perform DARTS assay. **B** Raw 264.7 cells were used to conducted ASM activity assay. Cells were treated in presence or absence of TNFα (10 ng/mL) overnight, then lysed by freeze-thaw in liquid nitrogen for three cycles, followed by collecting supernatants after centrifuging for activity assay. **C–J** Flow cytometry to test effects of penfluridol on spleen naïve CD4+T cell differentiation into T cell subsets. **C** Differentiation from naïve CD4+T to TH1. **D** Quantification of **C**. **E** Differentiation from naïve CD4+T to TH2. **F** Quantification of **E**. **G** Differentiation from naïve CD4+T to TH17. **H** Quantification of **G**. **I** Differentiation from naïve CD4+T to Treg. **J** quantification of **I**. Experiments were performed for 3 biological replications and one-way analysis of variance and Bonferroni post hoc test were used to test statistical significance of the differences among groups. (NS = not significance, *p < 0.05, **p < 0.01, ***p < 0.001, ****p < 0.0001). PF: penfluridol

The inhibitory effect of penfluridol on TNFα-induced NF-κB activation and anti-inflammation effects promoted us to explore the molecular mechanisms underlying penfluridol’s anti-TNFα activity. For this purpose, we first examined whether penfluridol affected the binding between TNFα and TNFα receptors. We thus performed solid phase binding and flow cytometry and found that penfluridol did not impact the binding between TNFα and TNFα receptors (Sfig. 6 A-B). We next performed bioinformatics analysis which led to the identification of ASM as a potential target of penfluridol. We undertook several methods to test the association between penfluridol and ASM. Firstly, a DARTS assay was employed to determine the binding of penfluridol and ASM. DARTS results showed elevated protein level of ASM in the presence of penfluridol at the ratio 1:1600 of pronase versus protein, indicating that penfluridol bound to ASM and protected ASM from pronase-mediated digestion (Fig. 6A). Secondly, we assessed the ASM activity to test whether the binding of penfluridol to ASM would affect the ASM activity and found that ASM activity was significantly inhibited by penfluridol (Fig. 6B). As ASM activity could be enhanced by inflammatory cytokines, such as TNFα, we further investigated whether TNFα elevated ASM activity could be suppressed by penfluridol. Results showed ASM activity was significantly enhanced by TNFα, which were obviously inhibited by penfluridol in a dose-dependent manner (Fig. 6B). However, western blotting analysis revealed that penfluridol did not affect the expression of ASM in the presence or absence of TNFα stimulation (Sfig. 7 A-B). Collectively, penfluridol could inhibit the ASM activity through direct binding to it.

Given that ASM was the target of penfluridol and penfluridol could inhibit the production of inflammatory cytokines induced by TNFα, we set to investigate whether the inhibitory effects of penfluridol on TNFα-induced cytokine production depend on ASM. Thus, effects of penfluridol on mRNA expression and cytokine secretion level were detected after knocking down ASM using siRNA (Sfig. 8A). After ASM knockdown, the inhibitory effect of penfluridol on TNFα-induced mRNA expressions and secretions of inflammatory cytokines, including IL-1β, IL-6, and NOS-2, were largely lost, suggesting the inhibitory effects of penfluridol on TNFα-induced NF-κB activity was at least partially dependent on ASM (Sfig. 8 B-E). To sum up, ASM was a target of penfluridol, and penfluridol exerted an anti-inflammatory effect through binding to ASM and inhibiting the activity of ASM.

Previously published data reported that ASM played an important role in regulating the differentiation of naive CD4+T cell into T cell subsets [36, 37]. Since ASM was identified as a target of penfluridol, we tested whether penfluridol affected naive splenic CD4+T cell differentiation in vitro. Results showed penfluridol inhibited naive CD4+T cell differentiation into TH1 and TH17 dose-dependently while penfluridol did not affect the differentiation of CD4+T cells to TH2 and Treg (Fig. 6C–J).

## Discussion

Penfluridol was isolated as a small-molecule drug that could inhibit TNFα-induced NF-κB activation and production of inflammatory cytokines. Further study found ASM was the target of penfluridol and demonstrated penfluridol could inhibit naive CD4+T cell differentiation to TH1 and TH17. Previously published reviews have suggested that ASM could mediate activation of NF-κB stimulated by TNFα [38–40]. Specifically, after TNFα binding to TNFα receptor 1 (55 kDa), phosphatidylcholine-specific phospholipase C is activated, which hydrolyzes phosphatidyicholine to produce 1,2-diacylglycerol, the well-known second messenger which is a activator of the ASM, and after the activation of ASM, it can regulate the activation of NF-κB through the production of ceramide which might affect proteolytic degeneration of IκBα. Thus, the NF-κB signaling pathway is activated, and a positive feedback loop is established to induce the production of the inflammatory cytokines TNFα, IL-1β, IL-6, and IL-23, which on the one hand promote the production of more inflammatory cytokines and on the other hand affect T cell differentiation and macrophage polarization. Accordingly, inhibiting the TNFα-stimulated NF-κB signaling pathway exerts anti-inflammatory effects and can inhibit the differentiation of naïve CD4+T cells.

Inflammatory cytokines are released by a wide range of immune cells, such as macrophages and T cells, and play a role in regulating both naive CD4+T cell differentiation and macrophage polarization. For instance, IL-1β and IL-6 are involved in the differentiation of naive CD4+T cell to TH17, and TNFα, IL-1, and IL-6 can promote the macrophage polarization to M1 [35, 41, 42]. In addition, TNFα, IL-1β, and IL-6 secreted by macrophages were reported to induce the expressions of the transcription factor T-bet and RORγt in T cells, leading to the differentiation from naïve CD4+T cells to TH1 and TH17 cells [43].

Penfluridol was reported as a functional inhibitor of ASM by Kornhuber in 2011 [44, 45]. To investigate the inhibitory effects of penfluridol on ASM, we endeavored to elucidate whether penfluridol bound to ASM by bioinformatics analysis, and results suggested their binding, which was further confirmed by DARTS assay. These results provide evidence that penfluridol might be used to treat conditions associated with increased ASM/ceramide levels, like Alzheimer’s disease, major depression, radiation- and chemotherapy-induced apoptosis, and endotoxic shock syndrome. Nevertheless, longer-term use of penfluridol may cause off target effects as it has been reported by recent studies that penfluridol could induce apoptosis, suppress Akt and mitogen-activated protein kinase activities, and augment intracellular ROS levels [46–48].

Several limitations have been also noted for this study. Firstly, more dosages and frequencies, including the lower dose group (0.4 mg/kg), for the treatment of arthritis need further investigations for the follow-up preclinical studies. Secondly, for the in vivo study, we only adopted firstline drugs, MTX and 5-ASA, as the positive controls, considering they are all small-molecule drugs. Whether the treatment effect of penfluridol is comparable to other relevant inhibitors, such as Enbrel and Humira, which inhibit NF-κB activation as biologics, needs further study. Thirdly, the CD4+T cells isolated from spleen by naive CD4+T isolation kit may only have naive CD4+T cells, whether penfluridol has the same inhibitory effect on memory CD4+ T cells as on naïve CD4+T cells also needs further study. Finally, whether ASM is required for the activation of NF-κB is contradictory. Previous studies reported that TNFα could activate NF-κB by ceramide produced by ASM hydrolyzing sphingomyelin in immune cells [40] whereas Kuno 1994 and Zumbansen 1997 et al. [49, 50] suggested TNFα could activate NF-κB in ASM-deficient Niemann-Pick disease type A fibroblasts and ASM knockout embryonic fibroblasts, suggesting the activation of NF-κB is through the canonical signaling pathway without relying on the ASM. Nevertheless, our study showed the inhibitory effect of penfluridol on cytokine production was lost after knockdown ASM in Raw 264.7 macrophages, suggesting the cytokine production induced by TNFα partially relied on ASM. This may be explained by the fact that different cell types were used in these studies and this paradoxical controversy warrants further studies.

## Conclusions

To sum up, penfluridol is a newly found drug that can inhibit TNFα-induced NF-κB activity by targeting ASM that penfluridol binds to, thus reduces ASM activity, which not only provides further understanding of the treatment mechanism for penfluridol but also broadens new possible applications of penfluridol to autoimmune diseases and elevated ASM activity-related diseases, in addition to the well-known role in treating chronic schizophrenia.

## Supplementary Information

**Additional file 1.**

**Additional file 2: Sfig. 1** Quantification of western blotting. **Sfig. 2** Penfluridol decreases cytokine expression and secretion induced by TNFα in Raw 264.7 cells. **Sfig. 3** Penfluridol decreases cytokine expression and secretion in hTNF-TG BMDMs. **Sfig. 4** Penfluridol inhibits osteoclastogenesis and macrophage polarization. **Sfig. 5** mRNA expression levels of CXCL10 and MCP-1 are decreased in penfluridol treated mouse models. **Sfig. 6** Penfluridol does not affect binding of TNFα to the receptors. **Sfig. 7** Effect of Penfluridol on ASM expression detected by western Blotting. **Sfig. 8** Effect of penfluridol on inflammatory cytokine production after knock down ASM. **Supplementary Methods**.

## Data Availability

All data are presented in tables or can be found in supplementary documents.

## Abbreviations

TNFα: Tumor necrosis factor alpha
RA: Rheumatoid arthritis
IBD: Inflammatory bowel disease
TNFi: TNFα inhibitors
ACR50: American College of Rheumatology standard
hTNF-TG: Human TNFα transgenic
WT: Wild-type
DMEM: Dulbecco’s modification of Eagle’s medium
FBS: Fetal bovine serum
TGF: Transforming growth factor
DSS: Ddextran sulphate sodium salt
TNBS: 2,4,6-Trinitrobenzenesulfonic acid solution
ELISA: Enzyme-linked immunosorbent assay
qRT-PCR: Real-time quantitative polymerase chain reaction
IL-1β: Interleukin-1 beta
NOS2: Nitric Oxide Synthase 2
BMDM: Bone marrow-derived macrophages
M-CSF: Macrophage colony-stimulating factor
RANKL: Receptor activating NF-κB ligand
CIA: Collagen-induced arthritis
5-ASA: 5-Aminosalicylic acid
MTX: Methotrexate
UC: Ulcerative colitis
CD: Crohn’s disease
