# Supplementary material for: Penfluridol targets acid sphingomyelinase to inhibit TNF signaling and is therapeutic against inflammatory autoimmune diseases
Source: Arthritis Res Ther. 2022 Jan 19;24:27. doi: 10.1186/s13075-021-02713-6 (PMC8767691; doi:10.1186/s13075-021-02713-6)
Supplement: Supplementary file 2 — Additional file 2: Sfig. 1 Quantification of western blotting. Sfig. 2 Penfluridol decreases cytokine expression and secretion induced by TNFα in Raw 264.7 cells. Sfig. 3 Penfluridol decreases cytokine expression and secretion in hTNF-TG BMDMs. Sfig. 4 Penfluridol inhibits osteoclastogenesis and macrophage polarization. Sfig. 5 mRNA expression levels of CXCL10 and MCP-1 are decreased in penfluridol treated mouse models. Sfig. 6 Penfluridol does not affect binding of TNFα to the receptors. Sfig. 7 Effect of Penfluridol on ASM expression detected by western Blotting. Sfig. 8 Effect of penfluridol on inflammatory cytokine production after knock down ASM. Supplementary Methods. [file 13075_2021_2713_MOESM2_ESM.docx]

**Penfluridol targets acid sphingomyelinase to inhibit TNF signaling and is therapeutic against inflammatory autoimmune diseases**

Yue-hong Chen^1,2^, Rong-han Liu^1^, Ya-zhou Cui^1^, Aubryanna Hettinghouse^1^, Wen-yu Fu^1^, Lei Zhang^1^, Chen Zhang^1^, Chuan-ju Liu^1,3*^

^1^Department of Orthopaedic Surgery, New York University Grossman School of Medicine, New York, NY 10003, USA; ^2^Department of Rheumatology and Immunology, West China Hospital, Sichuan University, Chengdu 610000, China; ^3^Department of Cell Biology, New York University Grossman School of Medicine, New York, NY 10016, USA

This Supplementary Material file includes:

**Sfigs. 1 to 8** (Pages 2-9)

**Methods** (Pages 10-12)

**References** (Pages 13)


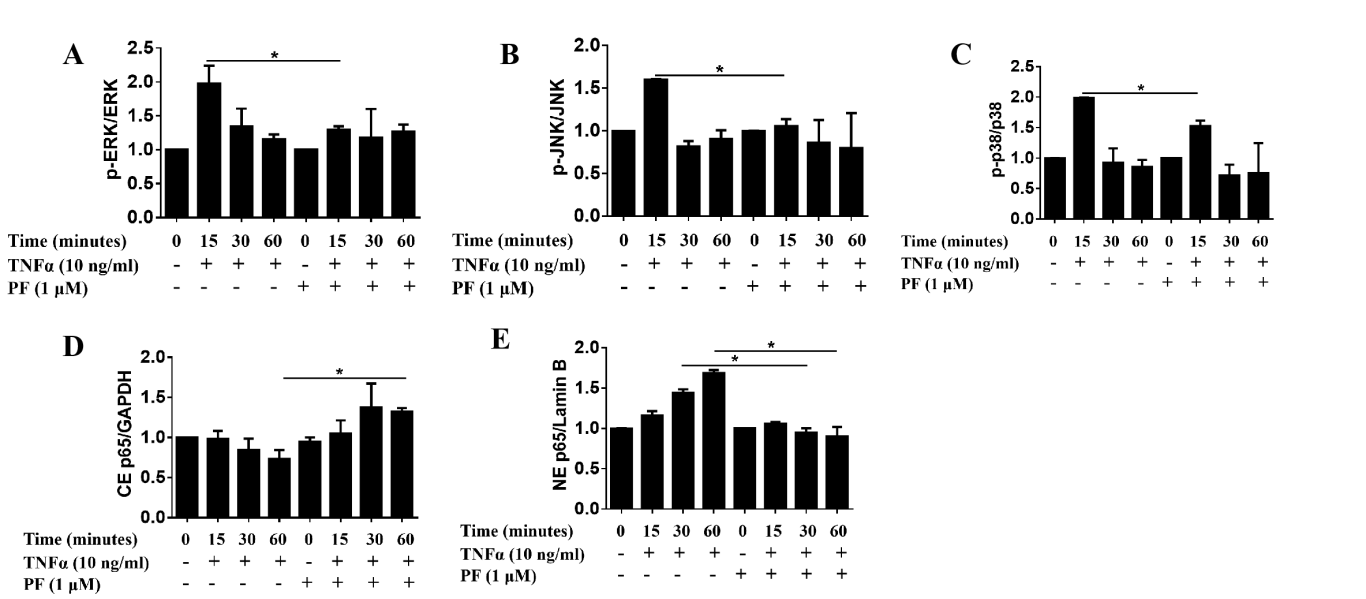


**Sfig.1 Quantification of western blotting.** BMDMs were maintained in DMEM supplemented with 2% FBS overnight, followed by adding DMSO or penfluridol (1μM) for 2 hours and then stimulated with TNFα (10ng/mL) for 0, 15, 30, or 60 minutes. A total of 20 μg protein samples were used to perform western blotting. (A-C) quantification of [phosphorylation](file:///C:/Users/apple/AppData/Local/Yodao/DeskDict/frame/20200720083644/javascript:void(0);) levels of ERK, JNK and p38 for figure 2A. (D-E) quantification of cytoplasmic extracts (CE) p65 and nuclear extracts (NE) p65 for figure 2B. Experiments were performed for 3 biological replications (*p<0.05).


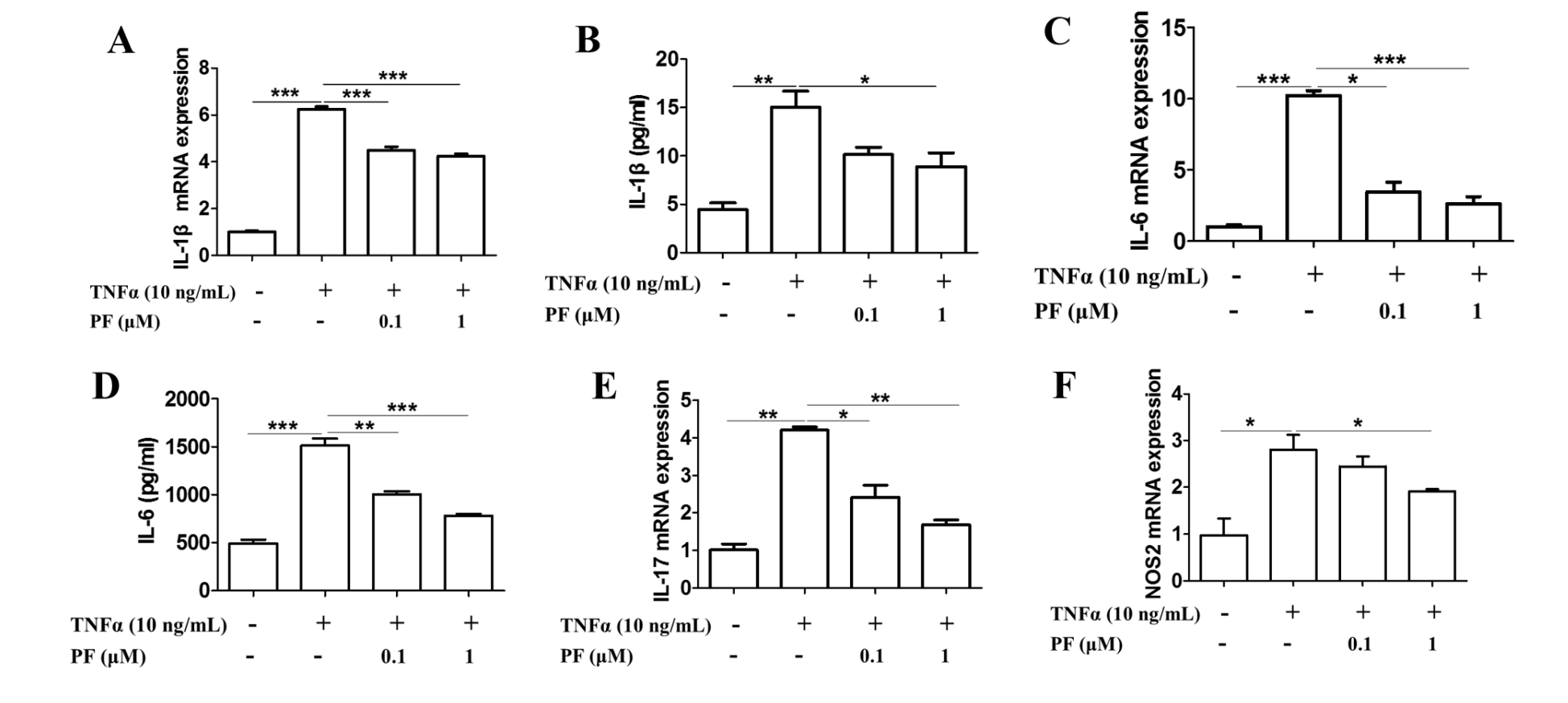


**Sfig.2 Penfluridol decreases cytokine expression and secretion induced by TNFα in Raw 264.7 cells**. Cells are treated with or without TNFα (10 ng/mL) and/or PF (0.1μΜ or 1μΜ) for 24 hours. (A, C) mRNA expression levels of IL-1β and IL-6 detected by qRT-PCR. (B, D) IL-1β and IL-6 levels in cell culture supernatants detected by ELISA. (E, F) mRNA expression levels of IL-17 and NOS-2 detected by qRT-PCR. Experiments were performed for 3 biological replications (*p<0.05, **p<0.01, ***p<0.001).


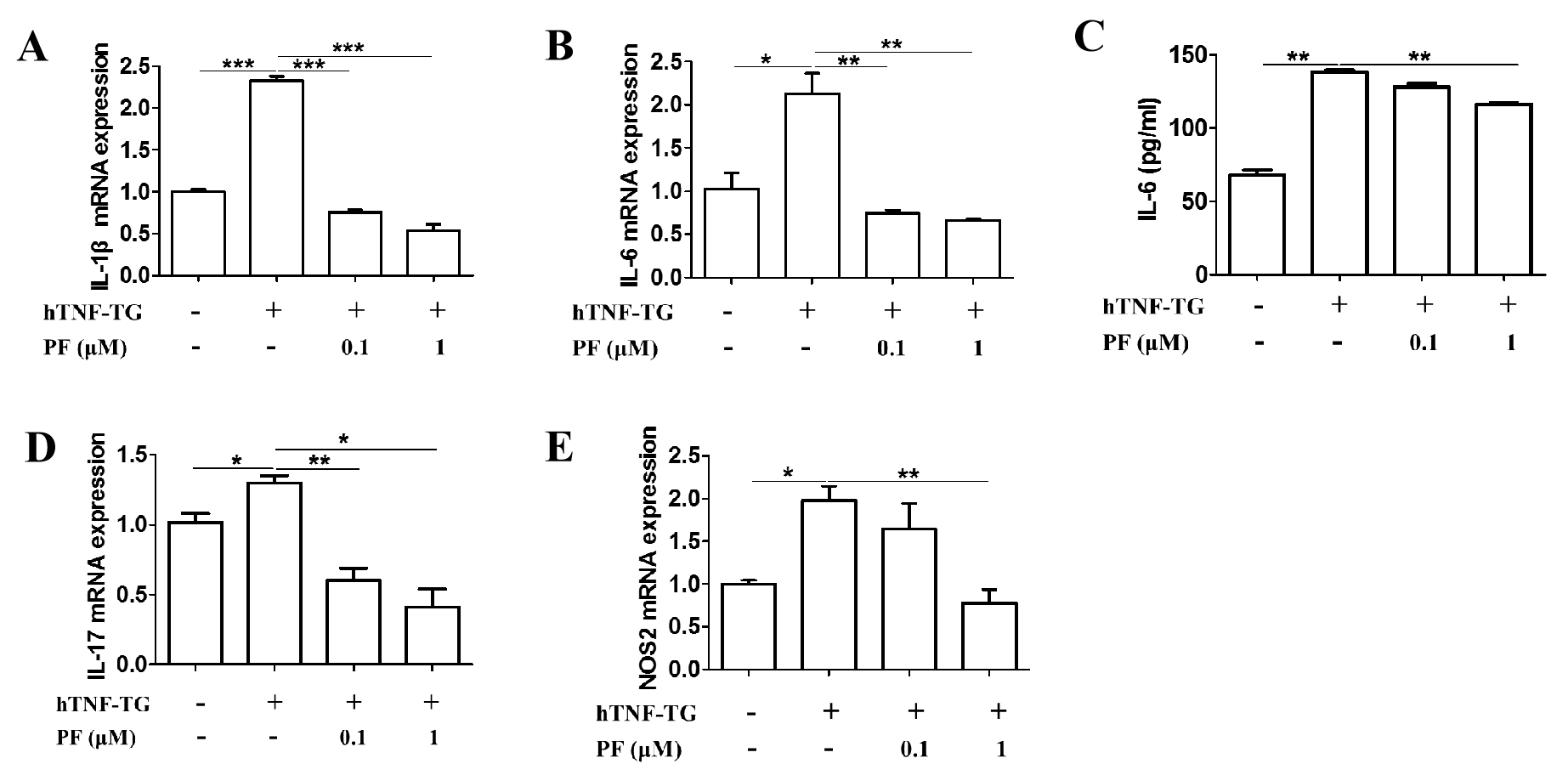


**Sfig.3 Penfluridol decreases cytokine expression and secretion in hTNF-TG BMDMs**. Cells are treated with or without PF (0.1μΜ or 1μΜ) for 24 hours. (A, B) mRNA expression levels of IL-1β and IL-6 detected by qRT-PCR. (C) IL-6 level in cell culture supernatants detected by ELISA. (D, E) mRNA expression levels of IL-17 and NOS-2 detected by qRT-PCR. Experiments were performed for 3 biological replications (*p<0.05, **p<0.01, ***p<0.001).


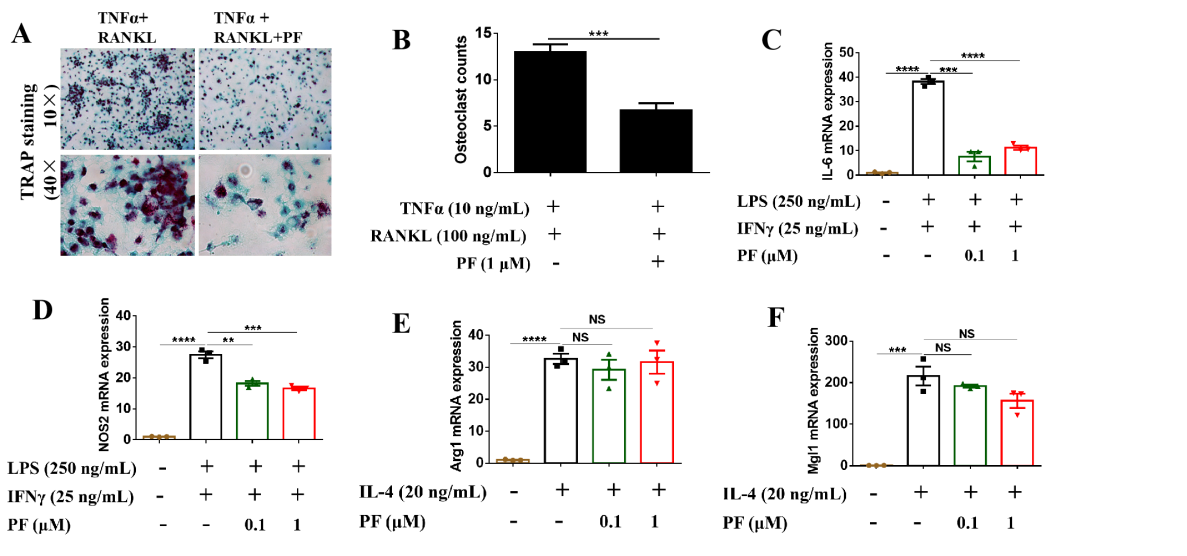


**Sfig.4 Penfluridol inhibits osteoclastogenesis and macrophage polarization.** Bone marrow cells were used to induced osteoclastogenesis by adding M-CSF (10 ng/mL), TNFα (10 ng/mL), and RANKL (100 ng/mL) in presence or absence of PF (1μM). (A) TRAP staining shows inhibitory effect of penfluridol on TNFα induced osteoclastogenesis. (B) Quantification of osteoclast number in A. (C-F) induction of macrophage polarization in BMDMs. qRT-PCR was used to test mRNA expression levels of macrophage molecular markers, IL-6 and NOS2 for M1 macrophage and Arg1 and Mgl1 for M2 macrophage. (C) IL-6 mRNA expression level. (D) NOS2 mRNA expression level. (E) Arg1 mRNA expression level. (F) Mgl1 mRNA expression level. Experiments were performed for 3 biological replicates (NS=not significance, **p<0.01, ***p<0.001, ****p<0.0001).


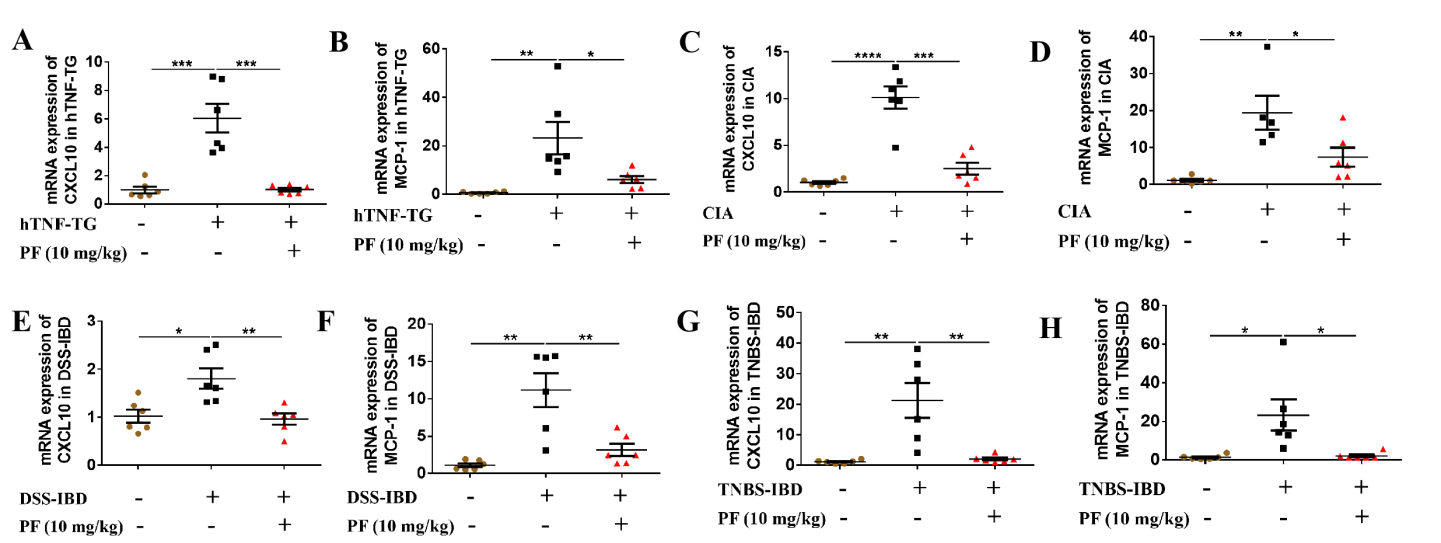


**Sfig.5 mRNA expression levels of CXCL10 and MCP-1 are decreased in penfluridol treated mouse models.** mRNA expression levels of CXCL10 and MCP-1 were detected by qRT-PCR in inflamed joints collected from hTNF-TG and CIA mice or colons harvested from DSS-IBD and TNBS-IBD mice. Six mice each group (*p<0.01, **p<0.01, ***p<0.001, ****p<0.0001).


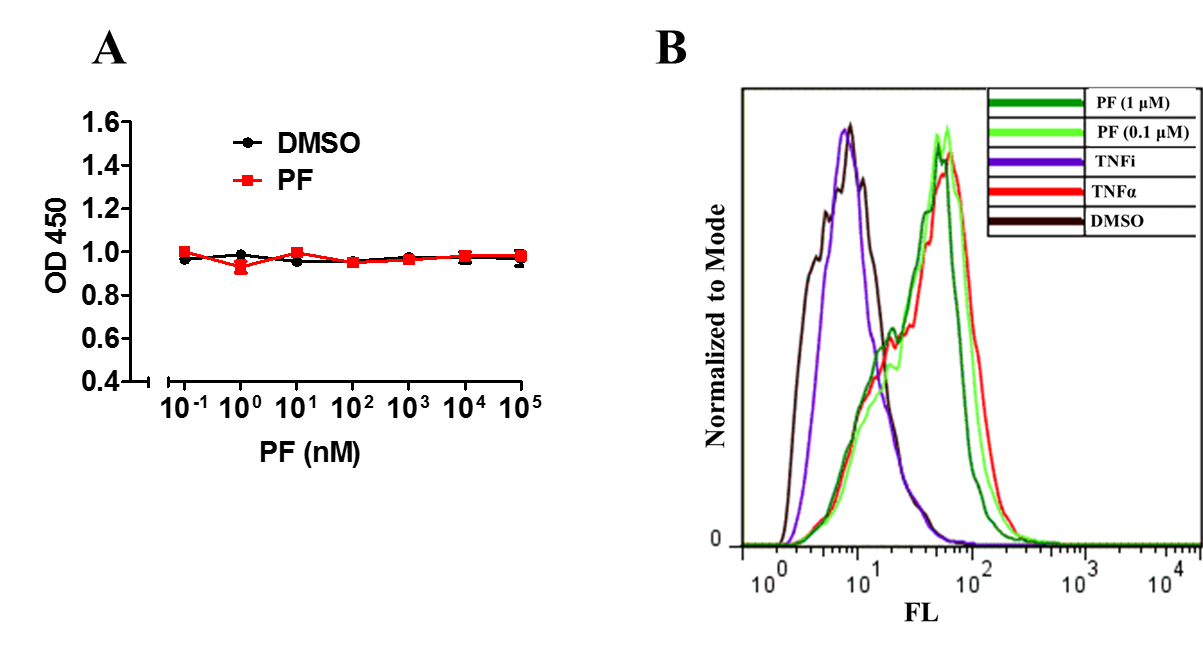


**Sfig.6 Penfluridol does not affect binding of TNFα to the receptors.** (A) Solid phase binding to test the binding between TNFα and TNFR1. (B) Flow cytometry in Raw 264.7 cells to test the binding between TNFα and receptors on the Raw 264.7 cell surface. Experiments were performed for 3 biological replicates.


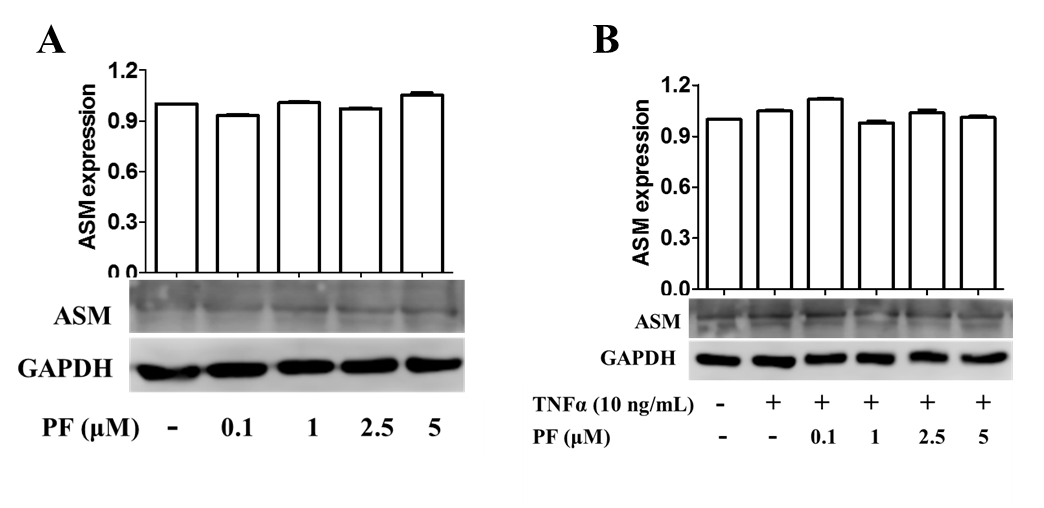


**Sfig.7 Effect of Penfluridol on ASM expression detected by western Blotting**. WT BMDMs are treated with or without TNFα (10 ng/mL) and PF (0.1μΜ, 1μΜ, 2.5μΜ, or 5μΜ) for 24 hours. (A) ASM expression without TNFα stimulation. (B) ASM expression with TNFα stimulation. ASM= acid sphingomyelinase. Experiments were performed for 3 biological replicates.


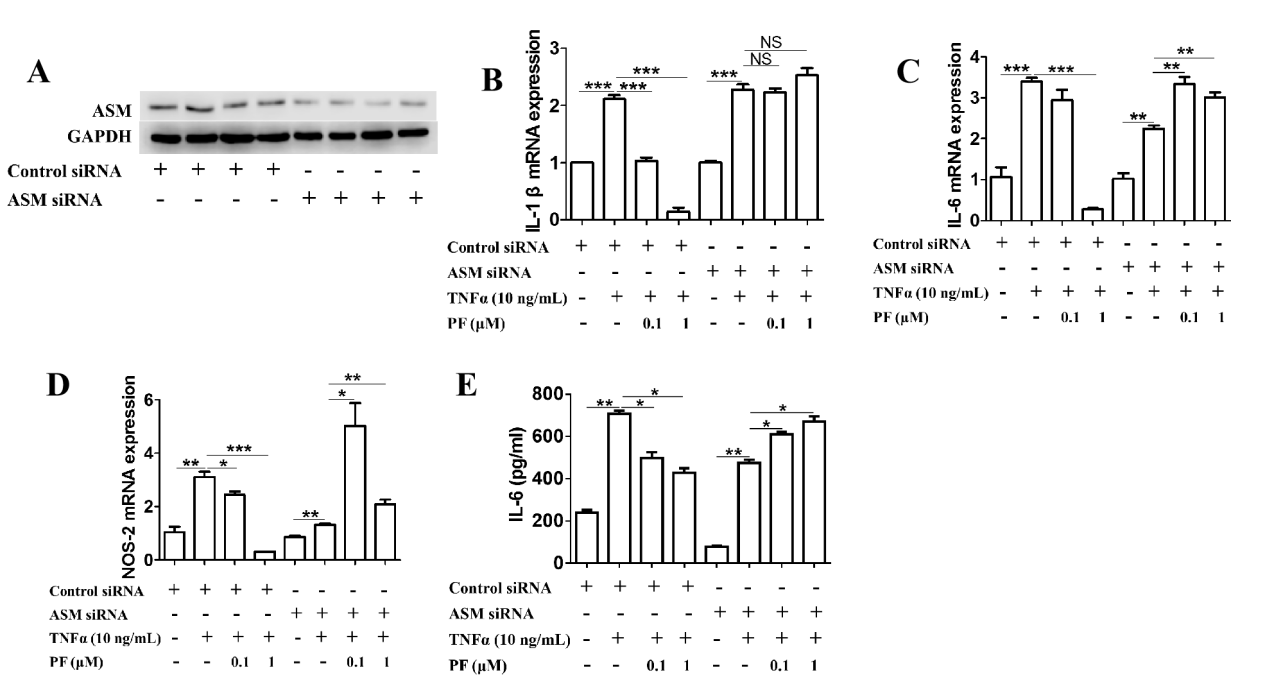


**Sfig.8 Effect of penfluridol on inflammatory cytokine production after knock down ASM.** Raw 264.7 cells were transfected with control siRNA (100pg) and si ASM (100pg) using lipofectamine 2000 for 24 hours. TNFα (10 ng/mL) was added with or without PF (0.1μM or 1μM) for 24 hours. (A) western blotting to detect ASM expression level after knocking down ASM expression by siASM. (B) qRT-PCR to detect IL-1β mRNA expression level. (C) qRT-PCR to detect IL-6 mRNA expression level. (D) qRT-PCR to detect mRNA expression level of NOS-2. (E) ELISA to detect secretion level of IL-6. Experiments were performed for 3 replications (NS=not significance, *p<0.05, **p<0.01, ***<p0.001).

**Supplementary Methods**

**TRAP staining**

Preparation for osteoclasts. WT C57BL/6 mice were sacrificed, and cells from the medullary cavity of femurs and tibias were collected and seeded in a 10cm dish. The next day, floated cells that were un-touched to the dish were seeded on a cover glass in 24-well plates and M-CSF (10 ng/mL) was added. After three days, cell culture medium was replaced with freshly made medium containing M-CSF (10 ng/mL), TNFα (10 ng/mL), and RANKL (100 ng/mL) with or without PF (1μM). Cells were allowed to undergo osteoclastogenesis for 4 days after which TRAP staining was performed to observe the effect of PF on osteoclastogenesis.

TRAP staining was performed according to the manufacturer’s instructions. Briefly, cells were fixed with 10% formaldehyde for 10 minutes at room temperature, followed by incubation with TRAP staining solution mix for 30 minutes at 37ºC and counterstaining with 0.02% fast green for 30 seconds. Coverslips were dehydrated through graded ethanols and cleared with xylene. The osteoclasts were stained with red violet in the green background. Images were captured using a ZEISS A.1 AxioScope microscope. Three images were randomly taken for each slide and number of osteoclasts in each image was counted. Data were shown by three replicates in each group from average counts of each slide.

**Macrophage polarization**

BMDMs were used to induce macrophage polarization. For M1 polarization, IFNγ (25 ng/mL) and lipopolysaccharide (250 ng/mL) were added and for M2 polarization, interleukin-4 (20 ng/mL) was added, in presence or absence of PF (0.1μM) or PF (1μM) for 24 hours. Total RNA were extracted by RNeasy Plus Mini kit. mRNA expression levels of *NOS2* and *IL-6* were used to measure M1 polarization while *Arg1* and *Mgl1* were applied to measure M2 polarization. The following specific sequences as qRT-PCR primers were used for the target gene amplification: Arg1 (5’-3’) F: TGC CAA AGA CAT CGT GTA CAT TG, R: CTT CCC AGC AGG TAG CTG AAG; Mgl1 (5’-3’) F: CAG ATC CGT ATC TGT CTG GAT C, R: AGG TGG GTC CAA GAG AGG ATG; NOS2 (5’-3’) F: TGT TAG AGA CAC TTC TGA GGC TC, R: ACT TTG GAT GGA TTT GAC TTT GAA G; IL-6 (5’-3’) F: TTC CAT CCA GTT GCC TTC TTG, R: AGG TCT GTT GGG AGT GGT ATC.

**Solid phase binding**

Solid phase binding assay was performed to test whether PF affects the binding between TNFα and TNFR1 based on previously described methods [1]. Briefly, 100 μL of TNFR1 (0.5 ng/μL) in phosphate buffered saline (PBS) was coated in 96-well high-binding plate and incubated at 4 degree overnight. The plate was washed 5 times with PBS-Tween (PBST) and 300 μL of blocking buffer was added to each well for one hour incubation at room temperature. Discarding blocking buffer, 50 μL buffer containing 10-fold dilutions of PF at concentrations ranging from 0.1nM to 10^5^ nM was added to the plate and incubated at room temperature for one hour. Negative and positive controls were established by addition of BSA or TNFα, respectively, to triplicate wells. 50 μL buffer containing 10 ng biotin-labeled TNFα was added to each well, and incubated the plate at room temperature for 2 hours. After wash, 100μL buffer containing streptavidin-HPR (1:250 dilution) was added and the plate was incubated at room temperature for 30 minutes. After wash, 100μL TMB buffer was added to each well and the reaction was stopped when color in the positive control group turned blue. The plate was read by automatic plate reader at 450nm.

**Binding of TNFα to cell surface receptors**

1×10^6^ Raw 264.7 cells were prepared for each sample. Cells were incubated with PF (0.1μM or 1μM) or DMSO at room temperature for 30 minutes and samples were stained according to the manufacturer specifications. Briefly, biotinylated TNFα was added to the samples and incubated at 4⁰C for one hour, followed by avidin-FITC staining at 4⁰C for 30 minutes. The cells were re-suspended in 0.2 mL 10 mM PBS at a concentration of 4×10^6^ cells/mL. Samples were analyzed on the Skirball FACSCalibur analyzer at NYU Medical Center core facilities.

**Knock down acid sphingomyelinase expression**

Raw 264.7 cells were transfected with control siRNA (100pg) and siASM (100pg) using lipofectamine 2000. After 24 hours, TNFα (10 ng/mL) was added with or without PF (0.1μM or 1μM) and cells were cultured for another 24 hours. Supernatants were collected to detect cytokine levels, cells were lysed to detect ASM expression by Western blot, and total RNA were extracted from cells to detect the mRNA expression levels of cytokines.

**References**

1. Tian Q, Zhao S, Liu C: **A solid-phase assay for studying direct binding of progranulin to TNFR and progranulin antagonism of TNF/TNFR interactions**. *Methods Mol Biol* 2014, **1155**:163-172.
